# Computational design of a soluble mimic of the outer membrane LPS transport protein LptD suitable for screening of antibiotics

**DOI:** 10.1002/pro.70626

**Published:** 2026-05-17

**Authors:** Wenzhao Dai, Wenxuan Hu, Matthias Schuster, Laetitia Rožić, Bernd Roschitzki, Oliver Zerbe

**Affiliations:** ^1^ Department of Chemistry University of Zurich Zurich Switzerland; ^2^ Functional Genomics Center Zürich (FGCZ) Zurich Switzerland; ^3^ Present address: Biozentrum University of Basel Basel Switzerland; ^4^ Present address: Institute for Protein Design University of Washington Seattle Washington USA

**Keywords:** antibiotics, Gram‐negative bacteria, LPS transport, protein design, protein–protein interaction inhibitor

## Abstract

Lipopolysaccharides (LPS) are the principal chemical component of the outer leaflet of Gram‐negative bacteria and constitute the first barrier of defense against foreign molecules. Inhibition of LPS transport presents a novel concept for antibiotic discovery, and components of the transport bridge are targets of antimicrobial peptides. LptD, a β‐barrel outer membrane protein, the terminal module of the Lpt transport bridge, however, remains largely unexplored as a drug target as its biosynthesis is complicated and screens against membrane proteins are challenging. Herein, we report a computationally designed, soluble *E. coli* LptD periplasmic epitope mimic, LptDm. We describe an efficient *in silico* design pipeline that includes verification of interactions of LptD mimics with the cognate ligands LptA and thanatin using nuclear magnetic resonance (NMR) and size‐exclusions chromatography (SEC) techniques. A small peptide library demonstrates that LptDm allows for selection of high‐affinity binders against LptD, rendering LptD accessible to modern drug discovery approaches.

## 1 INTRODUCTION

Life expectancy has dramatically improved during the first half of the 20th century, largely due to the availability of anti‐infectives. However, in the past 50 years, we have witnessed a significant slow‐down in the discovery of novel antibiotics and a rapid increase in the development of antimicrobial resistances (AMR). A recent analysis attributed 1.3 million deaths directly to AMR and approximately 5 million deaths associated with AMR in 2019 (Antimicrobial Resistance Collaborators 2022; GBD 2021 Antimicrobial Resistance Collaborators 2024). Models predict that this figure could rise to 10 million deaths per year by 2050, which would then exceed the mortality rate of cancer.

The development of antibiotics against novel targets tends to offer more sustainable relief from resistances as their mechanisms may not be fully established yet. Surprisingly, antibiotics against novel targets have not entered the market for more than 50 years. Compounds that interfere with outer membrane biogenesis of Gram‐negative bacteria have recently triggered interests as antibiotics (Moffatt et al. 2025; Theuretzbacher et al. 2023). The lipopolysaccharide transport (Lpt) system, a multiprotein complex consisting of at least eight proteins (LptA‐G, LptM, YedD), that transports lipopolysaccharides (LPS) from the inner membrane (IM) to the outer leaflet of the outer membrane (OM), presents such a novel target (Chen et al. 2025; Gennaris et al. 2025; Miyazaki et al. 2025; Ruiz et al. 2006; Sherman et al. 2018; Yang et al. 2023).

The first proof‐of‐principle study showing that the Lpt system is druggable came from the protegrin‐based macrocyclic peptide Murepavadin that binds to LptD, specifically in *P. aeruginosa*. Murepavadin disrupts LPS transport into the outer membrane, and thereby kills these bacterial cells (Srinivas et al. 2010; Werneburg et al. 2012). In recent years, additional compounds have been put forth that target different components of the Lpt system. One example is Zosurabalpin, a late clinical‐phase cyclic peptide developed by Roche against *A. baumannii* infections, targeting LptF, an inner membrane component of the Lpt transport system (Pahil et al. 2024; Zampaloni et al. 2024). Other examples are derivatives of thanatin, a defense peptide from the soldier bug (Fehlbaum et al. 1996). They bind with high affinity to the periplasmic *E. coli* Lpt component LptA (Vetterli et al. 2018) and display low MICs against carbapenem‐resistant enterobacterials (CRE) (Schuster et al. 2023).

Within the Lpt system, the terminal translocon protein LptD occupies a crucial position, as it mediates the final release and insertion of LPS into the outer membrane (Chen et al. 2025). The periplasmic β‐jelly‐roll domain of LptD binds to the next module of the bridge, LptA. In addition, the lipocalin YedD (also termed LptY) is required under conditions where Lpt bridge integrity is challenged as it binds to and thereby stabilizes the periplasmic domain of LptD (Gennaris et al. 2025).

Consistent with its essential role, LptD has emerged as a validated antibacterial target. To date, ligand discovery efforts against LptD have been limited to the β‐barrel domain of the LptDE translocon. Phage display‐based screening using the LptD β‐barrel identified multiple peptide binders but did not yield compounds with strong antibacterial activity (Allyjaun et al. 2025). The isolated soluble jellyroll domain of LptD might be sufficient for discovering inhibitors against the LptD‐LptA interaction but has not previously been exploited as a screening target. This lack of exploration is likely due to significant biochemical and technical challenges, including difficulties in purifying well‐folded soluble constructs of the LptD periplasmic domain and the inherent instability of this region.

Photo‐crosslinking and MS data indicated that thanatin also targets LptD from *E. coli*, although the exact binding site was not known (Fiorentino et al. 2021; Vetterli et al. 2018). At present, however, systematic optimization of thanatin‐based peptides toward LptD has been limited by the absence of suitable screening platforms for the LptD periplasmic domain.

Recent progress in computational protein design has dramatically improved the capability to design stably folded proteins (Koga et al. 2012; Kortemme 2024; Kuhlman et al. 2003). AI‐driven protein design methods are now capable of generating protein backbones (Watson et al. 2023), creating a suitable amino acid sequence that would assume that backbone structure (inverse folding) (Dauparas et al. 2022; Dauparas et al. 2025), and predicting protein structures with high confidence (Lin et al. 2023).

Here, we introduce a computationally designed, soluble, stabilized mimic of the functionally active periplasmic β‐jelly‐roll domain of *E. coli* LptD, termed LptDm. LptDm expresses at high yield, adopts the canonical jelly‐roll fold, and is stable at elevated temperatures. Importantly, LptDm represents a structurally and functionally validated mimic of the periplasmic domain of wild‐type LptD and forms stable contacts with LptA. Using a small peptide library based on the thanatin scaffold, we demonstrate that the mimic can be used for selections of binders from peptide libraries, providing a robust platform for drug screening against this essential component of the Lpt system.

## 2 RESULTS

The periplasmic domain of *E. coli* LptD is the only portion of LptD that forms interactions with LptA and is the proposed binding site of thanatin. Several attempts were made to produce different lengths of the jelly‐roll domain, with and without the N‐terminal helix and its disulfide bridges or as a fusion to LptA (see Table S4, Supporting Information). Unfortunately, all these constructs were unfolded, and no binding or folding upon binding was observed in the presence of LptA or thanatin. We suspect that the jellyroll requires additional stabilization from the C‐terminal β‐barrel domain to be rigidly folded and functional. Accordingly, the N‐terminal part of the jelly‐roll was stabilized by fusing it to a soluble, rigidly folded, fully designed motif, while leaving the N‐terminal β‐strands unchanged to allow for LptA binding.

### 2.1 Computational design of LptD mimics

**FIGURE 1:**
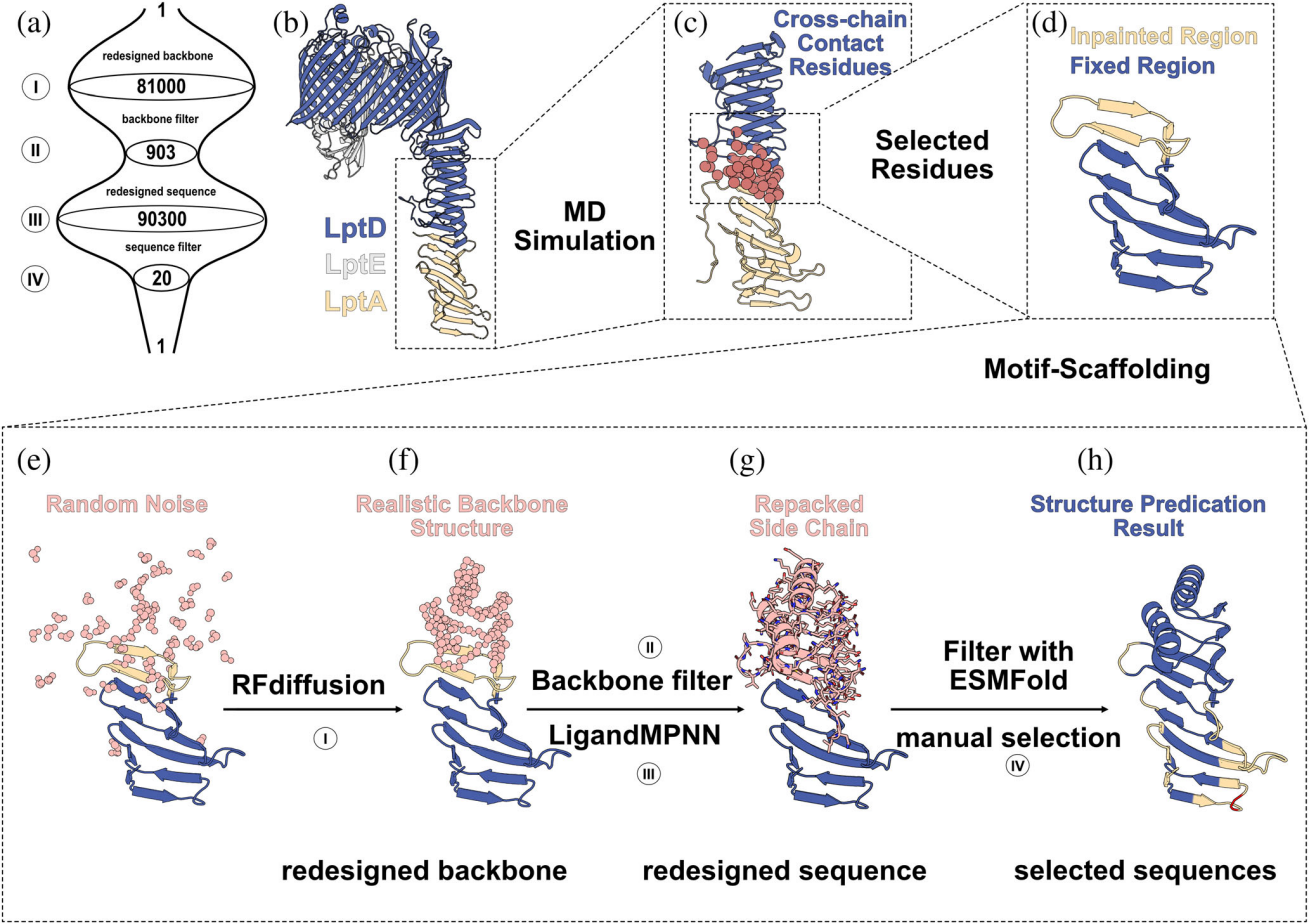
Computational design pipeline for soluble LptD mimics. (a) Number of candidates in each protein‐design step. (b) Model of the LptDE‐LptA complex from PDB entries 4Q35 and 2R19. Blue: wild‐type LptD, white: LptE, yellow: LptA. (c) Portion of the complex used for MD simulations. Residues of LptD that form contacts to LptA are depicted as red spheres. (d) Selected residues from LptD as fixed (blue) and inpainted (yellow) regions. (e, f) Result from motif‐scaffolding: blue: residues from LptD that are retained, yellow: inpainted residues, pink: residues generated by RFdiffusion. (g) Results from Inverse Folding, blue: preserved residues from wild type LptD, pink: residues redesigned by LigandMPNN, sticks: sidechain packed by LigandMPNN. (h) Protein structure from ESMFold with pLDDT ≤ 70 (red): 70 < pLDDT ≤ 90 (yellow), pLDDT > 90 (blue). pLDDT: predicted local distance difference test.

To stabilize the N‐terminal jelly‐roll of LptD while retaining its capability to recognize and bind cognate ligands, we applied an indirect motif‐scaffolding strategy (Figure 1a). An MD simulation of the LptD‐LptA complex (based on the structures of LptD (4Q35) and the LptA dimer (2R19)) identified all β‐strands in the segment G45‐D132 to contain residues that form direct contacts with LptA. Consequently, backbone and side‐chain conformations were retained in the respective region, which we termed *fixed* region. The neighboring 21–35 C‐terminal residues were termed *inpainted* region. This region forms stabilizing hydrogen bonds with the β‐strands of the fixed region. The stability of the inpainted region was reinforced by retaining the backbone conformation while altering the side chains in order to better stabilize the underlying hydrogen‐bond partners. Finally, a fully designed rigidly folded C‐cap, called *de novo* region, was added. In this architecture, the designed region supports the inpainted backbone, which in turn preserves the conformation of the fixed region.

RFdiffusion was applied to generate the designed region with 40–99 residues. Filters removed backbone candidates that display contacts between the designed and the fixed region, allowing a total of 903 out of 81,000 candidates to pass. LigandMPNN then created 100 sequences for the designed and inpainted regions of each of these designs. Structure predictions using ESMFold helped to identify promising candidates, and 20 candidates were finally tested by biophysical methods.

### 2.2 LptDm candidate selection

The C‐terminal modified jelly‐roll constructs were termed LptDm in analogy to the C‐terminal truncated LptAm construct. For LptDm to be functional it must display binding affinity to its native cognate protein LptA. Consequently, LptDm constructs were subjected to binding assays toward mLptA (Figure S2), the N‐terminally mutated LptA version, in analogy to the functional LptAm‐mLptA dimer (Schuster et al. 2023). For that purpose, the mLptA construct was stabilized in the first beta‐strand with LigandMPNN which lowered its tendency to oligomerize. With this construct, more accurate affinities for the mLptA‐LptAm and mLptA‐LptDm interactions were obtained (see Table S2 and Figure S8).

**FIGURE 2:**
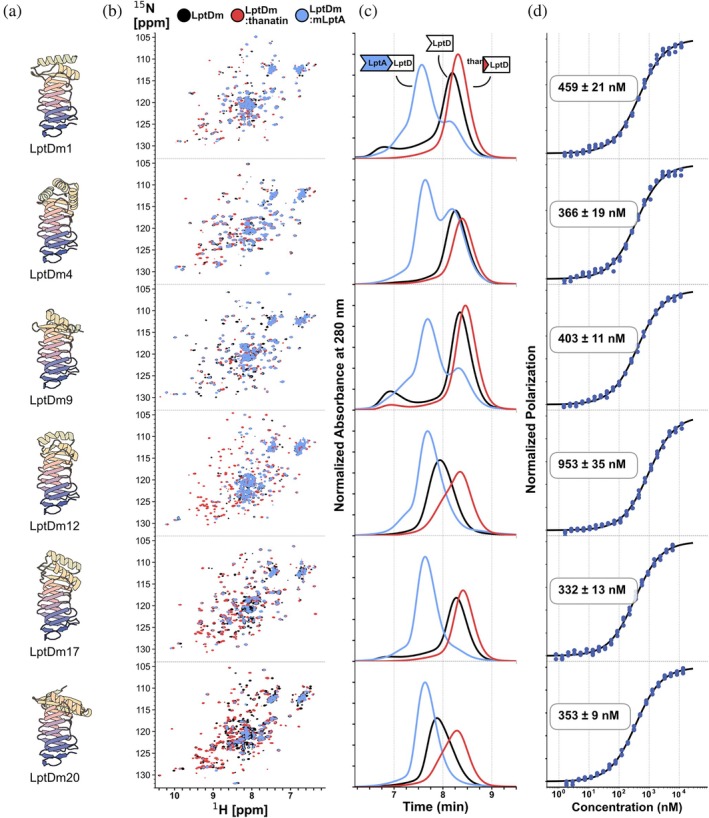
Characterization of selected LptDm mimics. (a) Structure designs of selected LptDm constructs. (b) Overlay of [^15^N,^1^H]‐HSQC spectra of apo‐state LptDm mimics (black), LptDm:thanatin complexes (blue), and LptDm:mLptA complexes (red). (c) Corresponding traces from the SEC. (d) *K*_d_s of each LptDm mimic to FL‐thanatin as directly determined by FP.

To rapidly decide which constructs to follow‐up, ^15^N‐^1^H correlation NMR spectra and size‐exclusion chromatography (SEC) elution profiles of the proteins were analyzed. Unfolded constructs were identified in the [^15^N,^1^H]‐HSQC spectra by their narrow proton chemical‐shift dispersion, whereas well‐folded constructs exhibited well‐dispersed cross‐peaks across the proton dimension (see Figures S1 and S7). SEC elution profiles revealed whether the protein was monomeric (Figure S6). In addition, ligand binding to mLptA and thanatin was investigated by monitoring chemical‐shift perturbations (CSPs) in [^15^N,^1^H]‐HSQC spectra and changes in retention times in the SEC traces. According to these criteria, six constructs (LptDm1, 4, 9, 12, 17, and 20) were selected, and binding affinities were determined by fluorescence polarization to fluorescently labeled thanatin (FL‐thanatin) (Figure 2d).

All constructs, except for LptDm12, displayed affinities to FL‐thanatin in the range of 300–500 nM (see Table S2). From the six selected candidates LptDm9, 12, and 20 were excluded due to their higher tendency to oligomerize (Figure 2c). Likewise, LptDm4 was excluded because incomplete complex formation with mLptA was observed in the NMR and SEC data (Figure 2b,c). Finally, LptDm17 was selected for further biophysical characterization as it displayed a higher binding affinity toward thanatin compared to LptDm1 (Figure 2b). For simplicity, LptDm17 is referred to as LptDm from now on.

The binding affinities of LptDm to the ligands thanatin and LptA were measured using a fluorescence polarization competition assay with FL‐thanatin (Table S2 and Figure S8). LptDm binds to thanatin with a *K*_d_ of 32.4 ± 16.2 nM, which is similar to the wild‐type LptD (27.9 ± 6.3 nM), and to the previously described thanatin analogue **7** with a *K*_d_ of 30.8 ± 15.6 nM (Schuster et al. 2023). These affinities were all measured in a LDAO buffer. However, we saw significant differences in affinities using different buffers (see Table S2). To obtain the affinity for mLptA by fluorescence polarization, a fluorescently labeled LptDm (FL‐LptDm; see Table S1) was produced, yielding an affinity of 985 ± 124 nM in TWEEN buffer. To verify that LptDm interacts with mLptA via the proposed interface, a double‐modified construct (mLptAm) that combines both terminal modifications from mLptA and LptAm was used as a negative control. The mixture of FL‐LptDm and mLptAm displays only unspecific binding in the high μM range (Table S2 and Figure S8), verifying the proposed interaction interface.

### 2.3 Structural and functional characterization of LptDm

**FIGURE 3:**
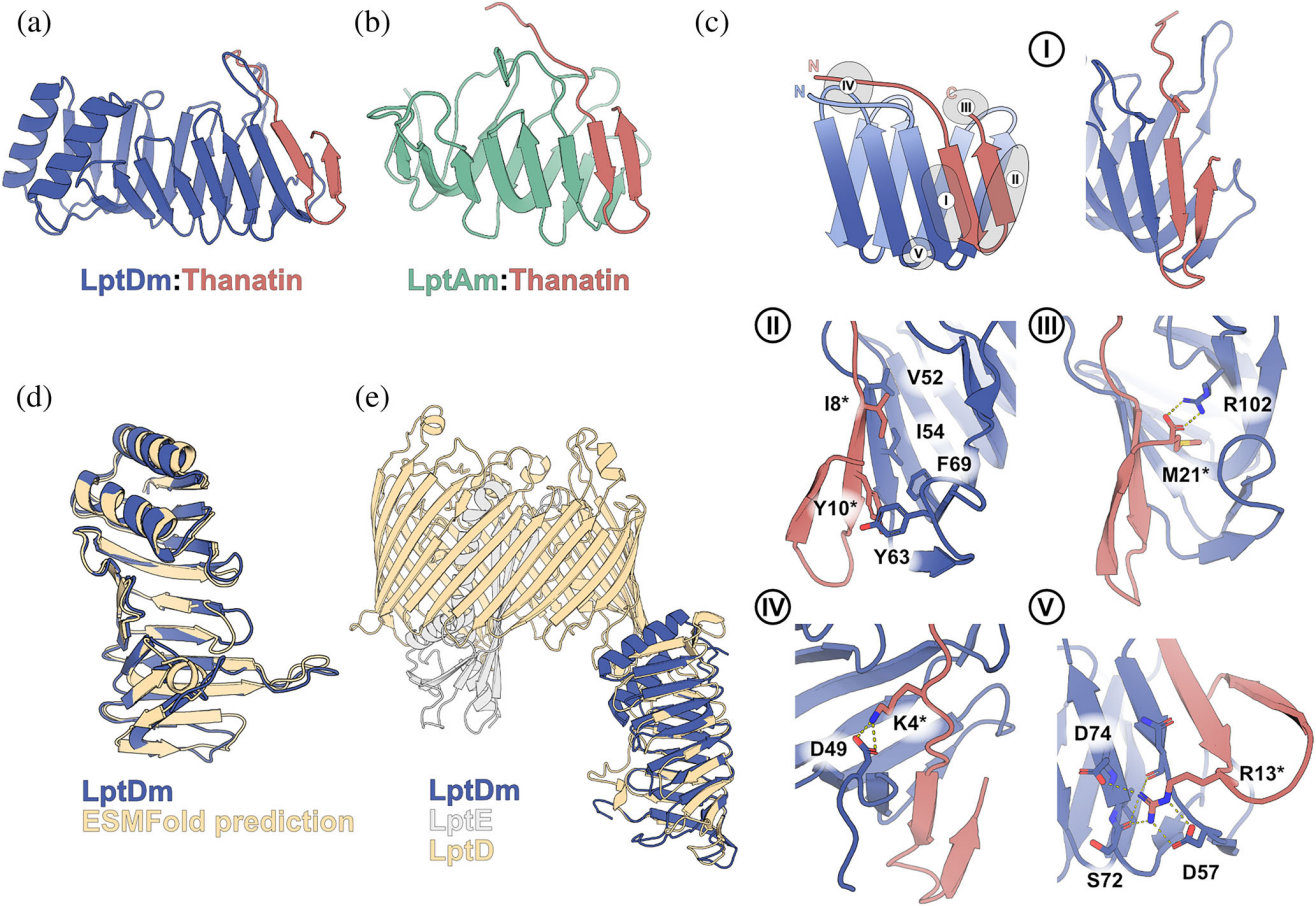
Analysis and comparison of LptDm:thanatin complex. (a) Lowest‐energy conformer of the LptDm:thanatin complex as determined by NMR (b) LptAm:thanatin structure PDB: 6GD5. (c) Structure details of the five major binding sites of the LptDm:thanatin complex. (d) Comparison of the NMR structure with the ESMFold prediction. (e) Comparison of the cryo‐EM structure (see Lehner et al., https://doi.org/10.21203/rs.3.rs‐8115403/v1) with the NMR structure.

A high‐resolution NMR structure (PDB: 9T3O) of the LptDm:thanatin complex was solved to a backbone‐RMSD of <1.0 Å (Figure 3a). The thanatin structure in the bound state was determined with a perdeuterated LptDm. The backbone and sidechain assignment of the LptDm:thanatin complex was performed with a ^13^C,^15^N labeled LptDm. Distance restraints were obtained from NOESY spectra (for further details of the structure determination see Table S5).

Both the native LptD fragment and the inpainted region adopt the jellyroll motif, while the de novo designed part incorporates a stabilizing helical hairpin. The binding interface of the LptDm:thanatin complex closely resembles that of the LptAm:thanatin complex (Figure 3b) (Schuster et al. 2023). In both complexes, thanatin binds in a parallel orientation via beta‐augmentation against the N‐terminal β‐strand of the jellyroll (Figure 3c(I)). These hydrogen‐bonds are further stabilized by hydrophobic sidechains that pack into the hydrophobic core of LptDm. Specifically, Ile‐8* of thanatin packs against Val‐52 and Ile‐54 of LptDm while Tyr‐10* (Figure 3c(II)) forms π‐stacking interactions with Phe‐69 and hydrophobic interactions with Tyr‐63 and Ile‐54 of LptDm (residue numbering refers to wild‐type LptD). Additionally, ionic interactions, although not always directly seen in the NMR conformers, appear stably formed during MD simulations (Figure S4). For example, Lys‐4* of thanatin interacts with Asp‐49 of LptDm, an interaction confirmed by NOE contacts between the two residues (Figure 3c(IV)). A similar interaction was observed for thanatin in its complex with LptAm (Schuster et al. 2023), and in that case involved Asp‐77 of LptAm. Moreover, Arg‐13* is associated with Asp‐57/Ser72/Asp74 (Figure 3c(V)) and the C‐terminal carboxy group of Met‐21* interacts with Arg‐102 of LptDm (Figure 3c(III)), two interactions that are conserved in the LptAm:thanatin complex.

The NMR structure of LptDm aligns closely with the ESMFold prediction, yielding a Cα‐RMSD of 1.62 Å. Furthermore, superposition of the LptDm:thanatin NMR complex with the corresponding residues in the LptDEM:thanatin cryo‐EM structure (using backbone atoms for thanatin residues 5–21 and LptD residues 51–91 + 102–132) reveals high structural homology with a Cα‐RMSD of 1.90 Å. Significant deviations are limited to the long loop (residues 92–101) and the side‐chain orientation of Tyr‐63 (see Figure S5). Ultimately, LptDm exhibits structural and functional features that closely mimic wt‐LptD binding thanatin with comparable affinity. It therefore serves as a reliable LptD mimetic for probing periplasmic interactions, i.e., during drug screening.

### 2.4 Peptide‐library screening against immobilized LptDm

To evaluate whether LptDm can be used for selections against a panel of different peptides, we performed a targeted peptide‐library enrichment. LptDm was C‐terminally biotinylated (LptDmAvi) and immobilized on streptavidin‐coated beads to serve as the selection bait. Based on the structure of the complex, it can be inferred that Y10* and M21* can be defined as critical anchor points, forming β‐strand contacts and hydrophobic interactions within the jelly‐roll. Therefore, we constructed a focused library involving systematic all‐residue substitutions at these positions (excluding Cys) (Table S3 red residues).

LC–MS/MS analysis of the retained fraction identified 198 unique peptides (FDR <0.5%) with the enrichment of sequences heavily biased toward hydrophobic residues at both Y10* and M21* (Table S3). While the top‐ranked hit (Peptide 1, M21Y) exhibited a *K*_d_ of 57.9 ± 8.3 nM, slightly higher than the parent thanatin (33.0 ± 4.2 nM), the recovery of nanomolar binders from a randomized pool confirms that the immobilized LptDm maintains a native‐like binding pocket. The slight discrepancy between MS‐based enrichment scores and equilibrium dissociation constants likely reflects the kinetic nature of the bead‐capture protocol and the intrinsic bias of MS detectability. However, the successful capture of a high‐affinity consensus motif demonstrates that LptDm is a viable platform for the identification of functional LptD ligands.

## 3 DISCUSSION

The rising problem of antimicrobial resistance urges innovative solutions in drug discovery. Most promising are drugs against novel targets for which resistance mechanisms are not yet fully developed. Modern target‐based drug‐development campaigns frequently start with screening large compound libraries against a newly discovered target (Terstappen et al. 2007). For validated targets such screens often rapidly identify successful lead compounds. The proteins of the LPS transport bridge indeed present such verified targets, as shown for Murepavadin (Andolina et al. 2018), thanatin derivatives (Schuster et al. 2023), and Zosurabalpin (Pahil et al. 2024; Zampaloni et al. 2024).

Given the high structural similarity of protein–protein interfaces in the Lpt bridge from various Gram‐negative bacteria, we recently suggested introducing an entirely new class of *narrow‐band* antibiotics against most interfaces based on a common thanatin scaffold (Schuster et al. 2023), including the LptD‐LptA interface. Complexes of new thanatin‐variants to *A. baumannii* LptA are structurally very similar to those of *E. coli* or *K. pneumoniae*. However, thanatin's lack of activity in *P. aeruginosa* or *A. baumannii* indicates that their exact chemical nature likely needs to be optimized for each pathogen (Oi et al. 2023). Thanatin additionally binds to LptD and therefore also might be optimized against that target.

However, when targeting the membrane protein LptD, technical issues significantly complicate screens for competitive inhibitors: Wild‐type LptD is difficult to express in functional form, and the requirement for its solubilization in a lipid‐mimicking environment often interferes with screening assays. Moreover, given the low sequence homology of LptD proteins of bacteria phylogenetically more distant to *E. coli,* such as *P. aeruginosa* or *A. baumannii*, we assume they do not bind thanatin with high affinity, requiring adaptations of the amino acid sequence. Those will be most easily identified when screening against large molecule libraries underlining the necessity for a simple high‐throughput screening procedure.

In this work we demonstrated that a protein comprising only the jelly‐roll domain suffices to select for compounds that tightly bind to the N‐terminal β‐strand of LptD in order to inhibit the LptD‐LptA interaction. Initial work on constructs corresponding to the periplasmic domain failed as they seem to lack crucial interactions with the beta‐barrel and are generally unfolded (see Table S4). Instead, we developed a computational design pipeline for converting the periplasmic domain of *E. coli* LptD into a soluble and stable LptDm periplasmic epitope mimetic that recognizes its natural ligands with similar affinities as the wild‐type protein and that is suitable for selections against compound libraries. To increase the stability of the isolated jelly‐roll domain, the beta‐barrel portion has been replaced with a soluble, rigidly folded domain. By combining modern computational protein design techniques with experimental screening, we engineered a stabilized soluble LptD mimic that is suitable for high‐throughput compound screens. We like to emphasize here, that LptDm is not a functional model of LptD, as it cannot transport LPS. However, it is an almost perfect structural mimic in its interaction with thanatin or LptA, as demonstrated by the binding affinities to thanatin and LptA that are very similar to wt‐LptDE, and because it neither requires LptM nor LptY for proper folding.

**FIGURE 4:**
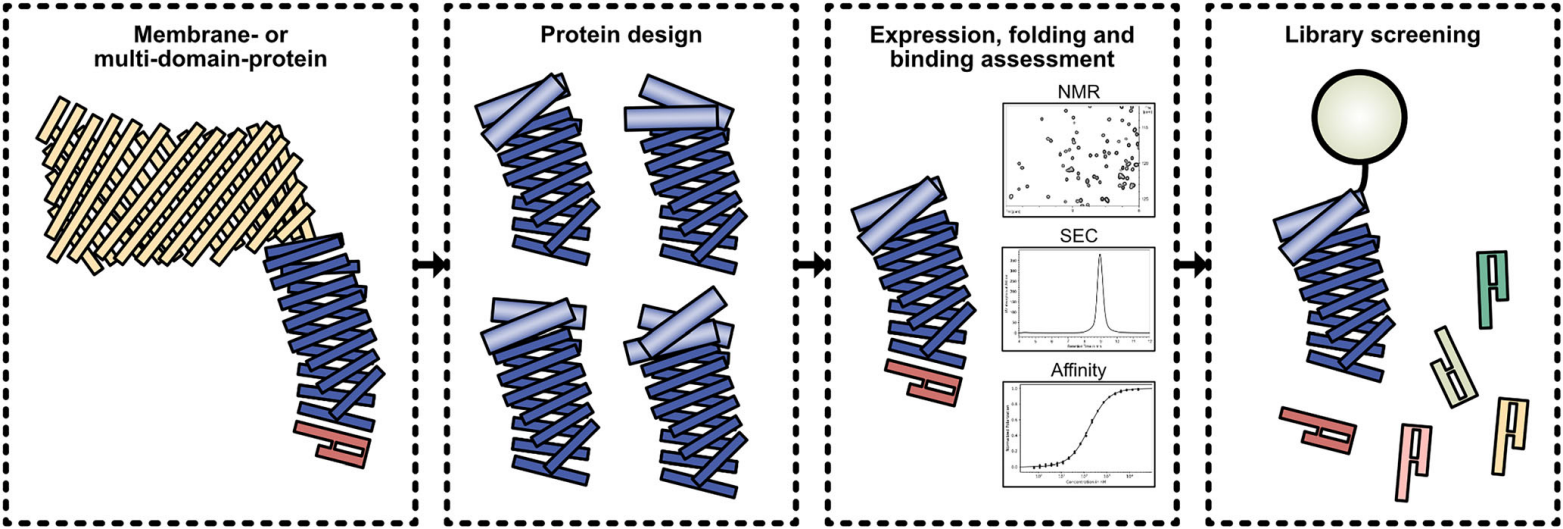
Scheme of workflow from in‐silico design to library screening (see text).

Our stabilized LptD mimic was developed via a highly efficient combination of *in silico* design and rapid validation of native‐like ligand binding (Figure 4). The approach facilitates the robust design of proteins with accurately grafted epitopes. SEC and heteronuclear NMR were used to select monomeric and folded constructs from the expressed designs and, even more importantly, to identify those that bind to the cognate ligands with high affinity. These checks are crucial to prevent wrong assumptions during the design process, allowing selections to be performed exclusively using mimics that recapitulate binding properties of the natural protein very well. The entire validation process for a single batch of designs was completed within one day after purification.

The *K*_d_ of the LptDm‐thanatin interaction is in the intermediate nM range and is similar as for wt‐LptD, suggesting neither the newly designed portion nor the missing beta‐barrel largely affect the interaction with binding partners at the N‐terminal site. The *K*_d_ between LptDm and mLptA is reported here for the first time as 985 ± 124 nM (all values in this paragraph in TWEEN buffer), which is about seven times weaker than the mLptA–LptAm interaction (*K*_d_ = 139 ± 4 nM) but comparable in strength to the previously reported LptAm–LptC interaction (*K*_d_ = 1.8 ± 0.4 μM) (Schuster et al. 2023). Thanatin binds roughly 9 times stronger to LptDm than mLptA to LptDm (122 nM vs. 986 nM), and thanatin binds 700 times stronger to LptAm than mLptA to LptAm (0.2 nM vs. 139 nM) (Table S2 and Figure S8). Therefore, LptD is only a secondary target of thanatin. This confirms the finding that thanatin is not active in the *E. coli* strain with a single LptA^Q62L^ mutation (Vetterli et al. 2018).

We like to note here that our NMR experiments prove that thanatin can disassemble the LptDm:mLptA complex (Figure S9). However, the lower affinity of thanatin to LptDm (122 nM compared to 0.2 nM for binding to LptAm in TWEEN buffer) indicates that thanatin will be primarily bound to LptA, and therefore LptA apparently is the biologically relevant target of thanatin. Nevertheless, considering that thanatin can disassemble the LptDm:LptA complex, we suspect that an optimized LptD‐binding peptide, based on the thanatin scaffold, might be developed that displays some orthogonality to the LptA binders. Such a peptide, when used in combination with LptA binders, could significantly reduce resistance rates.

The peptide **1**, the top‐ranked hit in the library, contained the M21Y mutation, similar to the previously developed thanatin analogue **7**. Interestingly, compound **7** also binds significantly better to LptDm (*K*_d_ 10 nM; see Table S2 and Figure S8). The binding of these peptides to LptD as a secondary target might have a positive influence on the MIC and the frequency of resistance and needs to be investigated in more detail in future studies.

During the development of thanatin‐based macrocyclic peptides directed against carbapenem‐resistant *Enterobacteriaceae* we observed that binding affinity to LptA was correlated to bioactivity, as measured by MICs in cultures of the bacteria of interest (Schuster et al. 2023). Accordingly, optimizing binding affinity to LptA was an important step in the discovery process. We assume that the success of the final optimization was related to the fact that with thanatin a nanomolar binder was already known at the start of the campaign. For other targets of the Lpt bridge and other pathogens, such high‐affinity binders are not available at present. Large screens using either recombinant or chemical libraries may promote a more rapid discovery of promising lead compounds. Due to this work, the N‐terminal LptD interface has become much more accessible to atomic‐level mechanistic studies as well as to high‐throughput drug screening campaigns. Therefore, we suggest that this novel approach will facilitate the development of antibiotics disrupting the Lpt bridge.

## 4 METHODS

### 4.1 Epitope verification by MD simulation

The Molecular Operating Environment (MOE) software was used to create a homology model of the LptD‐LptA heterodimer. Starting from the LptA dimer structure (2R19) the N‐terminal domain of the LptD structure (4Q35) was aligned with one of the LptA molecules. Additionally, the double disulfide bridge from the beta barrel to the N‐terminal jelly‐roll was retained, and everything else from the beta‐barrel was removed. The model was then energy minimized using the Amber force field and served as the starting structure for the MD simulation.

MD input files were then prepared using CHARMM‐GUI with the Solution Builder function and the CHARMM36m force field was used for proteins and ions, combined with the TIP3P water model, for the simulations. The N‐terminal domain of the LptD:LptA complex was placed in a rectangular water box with 10 Å padding distance. Periodic boundary conditions were applied in all directions, and long‐range non‐covalent interactions were treated with the Particle Mesh Ewald method. The system was equilibrated with 0.15 M NaCl at pH 7.

The MD simulation was performed using the OpenMM package. Prior to the production run, energy minimization was performed followed by NVT and NPT equilibrium at 310 K. The temperature was maintained at 310 K using Langevin dynamics. The production run was performed for 100 ns with a 2 fs time step saving the trajectory every ns for further analysis. Ten independent simulations were performed with random initial velocities. The trajectories were analyzed with the MDAnalysis package.

### 4.2 Motif‐scaffolding by RFdiffusion

RFdiffusion was used for motif‐scaffolding (Watson et al. 2023). Input residues were classified as “*fixed*,” “*inpainted*,” or “*de novo*” generated as described in the results section. The generated residues should make extensive contacts with the inpainted residues for stabilization, but avoid forming contacts to the fixed residues. RFdiffusion needed to be guided to generate backbones that fulfill these requirements. To this end two approaches were used.

RFdiffusion has sequence awareness and prefers to bury hydrophobic residues in the core while keeping charged residues at the surface. As a consequence, generated residues tend to contact hydrophobic residues but avoid contact with charged residues. To make use of this property, all fixed residues were changed to Glu, and all inpainted residues to Val before using RFdiffusion.

RFdiffusion supports a block adjacent matrix as part of the input to guide the contacts. This matrix function defines which fragments should or should not be close to each other. We created a custom adjacent matrix to enforce the proximity of generated residues with inpainted residues while avoiding contact with any fixed residue.

Additional backbone filtering from the RFdiffusion result was performed with PyRosetta. Backbones with more than 6‐residue loops were removed. Additionally, the radius of gyration (ROG) was calculated from the backbone atoms and candidates with ROG larger than 20 Å were eliminated. A contact map was calculated from the Cα atoms, and 8 Å was set as threshold for contacts between residues. Backbones with contacts between generated and fixed residues were removed. Backbone designs with less than 60 contacts between generated and inpainted segments were also filtered out. Contacts to residues 6 position upstream or downstream were defined as local contacts, all others were considered non‐local. Backbones with more than six continuous only‐local‐contact residues were removed.

### 4.3 Inverse folding by LigandMPNN

Subsequently, sidechains of the generated and inpainted segments were added to the backbone by LigandMPNN (Dauparas et al. 2025). The SolubleMPNN model trained with 0.20 Å Gaussian noise was used inside the LigandMPNN package. For each selected backbone, 100 sequences were generated. Cysteine residues were omitted in the sequence design.

### 4.4 Sequence filter

ESMFold was used for structure prediction for each LigandMPNN generated sequence (Lin et al. 2023). Each ESMFold output contained the pLDDT of the backbone atoms and the 5% quantile was defined as pLDDT_quantile5. USalign was used to calculate the tm_score, a measure of the agreement between the designed structure and the ESMFold prediction based on the sequence of the LigandMPNN output. Sequences with a pLDDT_quantile5 > 90 and a tm_score > 0.9 were selected for manual inspection.

### 4.5 Cloning, expression, and purification

All selected constructs were ordered as gBlocks from Twist Bioscience and cloned into a pEM3BT2 backbone vector via SapI and BamHI restriction sites (see Table S6). The LptA constructs were cloned via *in vivo* assembly (IVA). All constructs were expressed and purified as His_6_‐GB1‐*TEV*‐fusion proteins as previously described (Schuster et al. 2023).

The constructs were expressed in *E. coli* BL21 (DE3) cells at 25°C overnight upon 0.5 mM IPTG induction between OD_600_ 0.8–1.0 in ^15^N‐labeled M9 medium. Subsequent protein purification was performed on ice. The cell pellets (2–4 g) were resuspended in 15 mL Resuspension Buffer (20 mM Na‐phosphate pH 8, 300 mM NaCl, 10% glycerol, 5 mM MgCl_2_, 0.05 mg/mL deoxyribonuclease, 3 mg/mL lysozyme), then lysed by chemical lysis with 15 mL Lysis Buffer (80 mM Na‐phosphate pH 8, 300 mM NaCl, 10% glycerol, 2% Triton X‐100, 2% Na cholate, 40 mM imidazole, and 1 mM phenylmethylsulfonyl fluoride) for 1 h at 4°C while rolling. The lysate was clarified by centrifugation at 40,000*g* for 30 min. The resulting supernatant was purified with 2.5 mL of Ni‐NTA FastFlow resin. The columns were equilibrated with Lpt Buffer (20 mM Na‐phosphate pH 8, 300 mM NaCl, and 10% glycerol). Subsequently, the column was washed 2 × 20 mL of Lpt Wash Buffer (20 mM Na‐phosphate pH 8, 600 mM NaCl, 10% glycerol, 30 mM imidazole, and 20 mM Na‐cholate) and 2 × 20 mL with Lpt Buffer. The bound protein was eluted with 12.5 mL of elution buffer (20 mM Na‐phosphate pH 7.5, 300 mM NaCl, and 300 mM imidazole). The eluted protein fraction was treated with 1 mg of TEV protease, 1 mM EDTA, and 1/10 of cOmplete mini protease inhibitor tablet and was dialyzed overnight in 6–8 kDa cut‐off dialysis membrane against NMR Buffer (20 mM Na‐phosphate pH 7 and 150 mM NaCl) at 4°C. The Ni‐NTA column was equilibrated with NMR buffer and the protein was loaded onto the column 2 times and washed with an additional 2.5 mL of NMR buffer. The flow‐through was concentrated using an Amicon Ultra‐14 with a 10 kDa cutoff filter. The concentration of purified and concentrated constructs was measured by Nanodrop. The concentration of the protein for analytical SEC measurements and functional screening by NMR was 200 μM, 200 μM protein plus 350 μM thanatin, and 200 μM protein plus 200 μM mLptA.

### 4.6 *K*_d_ measurements by fluorescence polarization

LptDm containing an additional C‐terminal cysteine (LptDmc) was labeled with Alexa Fluor 647 maleimide. The protein was diluted to 1 μM in NMR buffer and supplemented with TCEP to a final concentration of 0.5 mM and incubated at 4°C for 1 h to reduce the thiols. TCEP was removed on a PD‐10 desalting column (Cytiva) that was pre‐equilibrated with NMR buffer. The elution was collected directly into a tube containing the Alexa Fluor 647 dye (Thermo Fisher Scientific, dissolved in 50 μL DMSO). The reaction was incubated for 2 h at room temperature in the dark. The unreacted dye was removed using a second PD‐10 column. The degree of labeling was verified by A280/A647 (~0.9) absorption using a nanodrop spectrometer.

All the protein–ligand and protein–protein binding affinities were determined by fluorescence polarization (FP). All proteins and ligands were dissolved in TWEEN FP Buffer (20 mM sodium phosphate (pH 7), 150 mM NaCl, and 0.05% TWEEN® 20 (Sigma‐Aldrich)), LDAO FP buffer (50 mM Tris (pH 8), 150 mM NaCl, and 0.1% LDAO (Sigma‐Aldrich)), screening buffer (20 mM sodium phosphate (pH 7.5), 150 mM NaCl), or BSA FP buffer (20 mM sodium phosphate (pH 7.5), 150 mM NaCl, and 0.05% BSA). All binding affinity assays were performed on Optiplate‐96F microplates (PerkinElmer) and measured in experimental triplicates. Protein–ligand binding affinities were measured either directly or via a competition assay. Both methods used a final well volume of 160 μL and a dilution series of 20 points with an initial concentration of 10–110 μM in the first well and subsequent dilution by 1.6‐ or 1.8‐fold increments. In the direct binding assays, fluorescently labeled ligand was added to reach a final concentration of 5 or 10 nM, while in the displacement assays, an additional protein was added to achieve a final concentration of 50–1100 nM. In general, FL‐thanatin was used for the displacement assays. The *K*_d_ values were calculated using the cubic equation described by Roehrl et al. (Roehrl et al. 2004) and using a python script with the lmfit module. The detailed FP measurement conditions were listed in Table S2.

### 4.7 Peptide library screening by LC/MS–MS

The thanatin peptide library was ordered from QYAOBIO as a crude extract where positions 10 and 21 were randomized to every amino acid except cysteine (Figure S3). To immobilize LptDm for the library selection, the protein was co‐expressed as an Avi‐tag fusion (LptDmAvi) in BL21(DE3) cells with a BirA‐pLEMO plasmid. Protein expression was induced with 0.5 mM IPTG, 1 mM L‐rhamnose monohydrate, and 100 μM biotin. The protein was purified as described above. The biotinylated LptDmAvi protein was subsequently saturated onto 500 μL Dynabeads™ MyOne™ Streptavidin T1 (allowing binding with a capacity of 2 nmol LptDm) to screen for peptide binders. The beads were washed 5 times with 1 mL screening buffer, 1000 nmol of peptide library was added in a total volume of 500 μL, incubated at 4°C for 1 h. Therefore, the protein should be saturated with the best binder. The beads were washed once with water and then eluted with 1% trifluoroacetic acid (TFA). The elution was analyzed via LC–MS/MS. To prepare the sample for LC/MS–MS the sample was reduced to break disulfide bonds and alkylated by adding dithiothreitol and chloroacetamide to final concentration of 10 and 15 mM, followed by 30 min incubation at 30°C with shaking at 800 rpm and light protection. Peptides were acidified and cleaned using in‐house packed C18 stage‐tips, loaded on tips and washed in 0.1% TFA in water, eluted with 0.1% TFA in 60% acetonitrile and then dried to completeness.

Peptides were redissolved in aqueous 0.1% TFA and separated using an M‐class UPLC system (Waters) and were analyzed on an Orbitrap mass spectrometer (Thermo Scientific). The resulting mass spectrometry data were processed for identification using PEAKS Studio X Plus (Bioinformatics Solutions). Spectra were searched against a protein sequence database, which includes the 361 synthetic peptides and the human proteome (UP000005640_1spg). The oxidation (M) and carbamidomethylation (C) were set as variable and fixed modifications, respectively. The precursor mass tolerance was set to 10 ppm and the fragment mass tolerance to 0.02 Da. Cyanobromide was defined as the cleavage reagent, and only fragments with up to two missed cleavages were allowed.

### 4.8 NMR spectroscopy, assignments, and structure calculation

The concentration of the LptDm (^15^N, ^13^C‐labeled) sample for structure determination was 1 mM plus 1.5 equivalents of thanatin. Peak picking, backbone assignment, and sidechain assignment were performed in CCPNmr3.2. Backbone assignment of LptDm was obtained from HNCA, HNCB, HNcoCACB, HNCO, HN(CA)O, and (H)N(CA)NNH spectra (Sattler et al. 1999). Sidechain assignment was performed using HBHA(CO)NH, H(C)CH‐TOCSY, and (H)CCH‐TOCSY spectra. Aromatic side chains were assigned using (HB)CB(CGCD)HD and (HB)CB(CGCDCE)HE and the aromatic ^13^C‐NOESY‐HSQC. The resonance assignment of thanatin was transferred from the LptAm:thanatin complex to the LptDm:thanatin complex using a ^12^C‐^14^N‐selected‐2D‐NOESY, recorded on fully perdeuterated, ^15^N, ^13^C labeled LptDm (600 μM plus 540 μM thanatin). This spectrum was also used to extract intramolecular distant restraints within thanatin. Intermolecular distant restraints were obtained from ^13^C‐^15^N‐filtered‐^13^C‐resolved NOESY spectra. In addition, distant restraints were extracted from ^15^N‐ and ^13^C‐resolved NOESY spectra recorded for both the aliphatic and aromatic regions. All NOESY experiments were recorded with a mixing time of 80 ms. The NOESY peak lists were automatically assigned using CYANA. To enforce correct assignment to intermolecular restraints the chemical shifts of thanatin were increased by 20 ppm in the chemical shift list and in the peak list of the ^13^C, ^15^N‐filtered‐^13^C‐resolved NOESY. Additional torsion angle restraints were derived from backbone chemical shifts using the program TALOS‐N.

The structure calculation was performed in CYANA with the standard parameters using 50,000 steps per simulated annealing cycle. The 20 lowest energy structures from 100 calculations were retained. An additional energy minimization in OpenMM 8.3 with the Amber ff19SB force field in a TIP3P water box was performed. For a summary of statistics of the structure calculation see Table S5.

## AUTHOR CONTRIBUTIONS

**Matthias Schuster:** Investigation; supervision; writing – original draft; writing – review and editing; visualization; formal analysis; data curation. **Wenzhao Dai:** Conceptualization; investigation; writing – original draft; methodology; validation; visualization; software; formal analysis; data curation; writing – review and editing. **Oliver Zerbe:** Funding acquisition; writing – original draft; writing – review and editing; project administration; supervision; resources; conceptualization. **Bernd Roschitzki:** Investigation; formal analysis. **Laetitia Rožić:** Investigation; writing – review and editing; formal analysis; data curation. **Wenxuan Hu:** Investigation; writing – review and editing; writing – original draft; visualization; methodology; formal analysis; data curation.

## CONFLICT OF INTEREST STATEMENT

The authors declare no conflicts of interest.

## Supporting information


**Table S1.** Amino acid sequence of all constructs.
**Table S2.** Binding affinities of Lpt proteins' candidates.
**Table S3.** Top scoring peptides from the library screening.
**Table S4.** List of LptD constructs that were unfolded and unable to bind thanatin or mLptA.
**Table S5.** Statistics from the NMR structure calculation.
**Figure S1.** Characterization of folding and thanatin‐binding by NMR.
**Figure S2.** The LC–MS spectrum of LptDm17.
**Figure S3.** The design of the peptide library and LC–MS/MS results of the pull‐down.
**Figure S4.** MD results of the lowest energy conformer of the LptDm NMR structure.
**Figure S5.** Structure comparison of the cryo‐EM structure of the *E. coli* LptDEM:thanatin complex with the LptDm:thantin NMR structure.
**Figure S6.** SEC chromatograms of all apo‐state LptDm variants.
**Figure S7.** Overlay of [^15^N, ^1^H]‐HSQC spectra.
**Figure S8.** Overview of all FP measurements.
**Figure S9.** [^15^N,^1^H]‐HSQC data demonstrating disassembly of the LptDm:mLptA complex upon addition of 2 equiv. of thanatin.
**Table S6.** List of Open Reading Frames (ORF) for LptD constructs.

## Data Availability

The data that support the findings of this study are openly available in Github at https://github.com/Wenzhao-protein/LptD_motif_scaffolding. Coordinates of the LptDm:thanatin complex have been deposited in the PDB repository under accession number 9T3O.
