# Supplementary material for: Computational design of a soluble mimic of the outer membrane LPS transport protein LptD suitable for screening of antibiotics
Source: Protein Sci. 2026 May 17;35(6):e70626. doi: 10.1002/pro.70626 (PMC13181209; doi:10.1002/pro.70626)
Supplement: Supplementary file 1 — Table S1. Amino acid sequence of all constructs. Table S2. Binding affinities of Lpt proteins' candidates. Table S3. Top scoring peptides from the library screening. Table S4. List of LptD constructs that were unfolded and unable to bind thanatin or mLptA. Table S5. Statistics from the NMR structure calculation. Figure S1. Characterization of folding and thanatin‐binding by NMR. Figure S2. The LC–MS spectrum of LptDm17. Figure S3. The design of the peptide library and LC–MS/MS results of the pull‐down. Figure S4. MD results of the lowest energy conformer of the LptDm NMR structure. Figure S5. Structure comparison of the cryo‐EM structure of the E. coli LptDEM:thanatin complex with the LptDm:thantin NMR structure. Figure S6. SEC chromatograms of all apo‐state LptDm variants. Figure S7. Overlay of [15N, 1H]‐HSQC spectra. Figure S8. Overview of all FP measurements. Figure S9. [15N,1H]‐HSQC data demonstrating disassembly of the LptDm:mLptA complex upon addition of 2 equiv. of thanatin. Table S6. List of Open Reading Frames (ORF) for LptD constructs. [file PRO-35-e70626-s001.pdf]

Supplementary Materials for

Computational design of a soluble variant of the outer membrane LPS transport protein LptD suitable for screening of antibiotics

Wenzhao Dai et al.

Content:

Table S1: Amino acid sequence of all constructs

Table S2: Binding affinities of Lpt proteins' candidates

Table S3: Top scoring peptides from the library screening

Table S4: List of LptD constructs that were unfolded and unable to bind thanatin or mLptA

Table S5: Statistics from the NMR structure calculation

Figure S1: Characterization of folding and thanatin-binding by NMR

Figure S2: The LC-MS spectrum of LptDm17

Figure S3: The design of the peptide library and LC-MS/MS results of the pull-down

Figure S4: MD results of the lowest energy conformer of the LptDm NMR structure

Figure S5: Structure comparison of the cryo-EM structure of the *E. coli* LptDEM:thanatin complex with the LptDm:thantin NMR structure

Figure S6: SEC chromatograms of all apo-state LptDm variants

Figure S7: Overlay of [^{15}N,^{1}H]-HSQC spectra

Figure S8: Overview of all FP measurements

Figure S9: [^{15}N,^{1}H]-HSQC data demonstrating disassembly of the LptDm:mLptA complex upon addition of 2 equiv. of thanatin.

Table S6: List of Open Reading Frames (ORF) for LptD constructs

Proteins	Sequences
**LptDm1**	GDTNDLPVTINADHAKGDYPDDAVFTGSVDIMQGNSRLQADEVQLHQKEAPG<br>QPEPVRTVDALGNVHYDDNQVILKGPKGWANLNTKDWKFEDASIEFVGMPGS<br>ASGKLIEYKNGTITIYIDENSKITEEFIEAATKLGYKVEVKGDEKVKEKVDELKEII<br>KKVEAK
**LptDm2**	GDTNDLPVTINADHAKGDYPDDAVFTGSVDIMQGNSRLQADEVQLHQKEAPG<br>QPEPVRTVDALGNVHYDDNQVILKGPKGWANLNTKDSVWYNSSVESVGAPGS<br>FSAEIILSLLNGKSLVAVDISEEDKPGFKEVAKNLGLKVFLETEVNGKKTLIASKD<br>EEDVPKALEKAEKIVKEVEKE
**LptDm3**	GDTNDLPVTINADHAKGDYPDDAVFTGSVDIMQGNSRLQADEVQLHQKEAPG<br>QPEPVRTVDALGNVHYDDNQVILKGPKGWANLNTKDSVWFDPSAEMVGLPGS<br>GSAKKIEFLDNGKLVKLVIDKEHYEKVKEELLKTVDAKVEELPDGSVAITTSGDP<br>ETVSKTLSILK
**LptDm4**	GDTNDLPVTINADHAKGDYPDDAVFTGSVDIMQGNSRLQADEVQLHQKEAPG<br>QPEPVRTVDALGNVHYDDNQVILKGPKGWANLNTKDATFWEASMEFVGAPG<br>AFTADEIKFLDNGKIVELILNGKTAKFLFPELLKTSKEAKEKGWSKEELIEAFKK<br>TLLPGSDAEVLEEAMKKA
**LptDm5**	GDTNDLPVTINADHAKGDYPDDAVFTGSVDIMQGNSRLQADEVQLHQKEAPG<br>QPEPVRTVDALGNVHYDDNQVILKGPKGWANLNTKDARFEDASMEFVGVPGS<br>ASATLIEKKDGAYFFKDPTFKDLSEELKELVLEADELLKKGDKKEALKKAYEFE<br>KLVAELLGNKEEVEKLEKKLKEV
**LptDm6**	GDTNDLPVTINADHAKGDYPDDAVFTGSVDIMQGNSRLQADEVQLHQKEAPG<br>QPEPVRTVDALGNVHYDDNQVILKGPKGWANLNTKDAVFEDASGEVVGLPGS<br>ASAKKIEFKSNGNIIVFYDVEDVEEAIKFAESYFKAQGKEVDLIIVKGKNVIASTE<br>EAKEEAKKIAEKI
**LptDm7**	GDTNDLPVTINADHAKGDYPDDAVFTGSVDIMQGNSRLQADEVQLHQKEAPG<br>QPEPVRTVDALGNVHYDDNQVILKGPKGWANLNTKDGVFYDASAENVGAPGS<br>ASAKKIEFLNNGKILVFYDATSSEEDVKTLAENYLKQLGEEAVFESIDGNFIAAA<br>KSKKAELEKLAAELK
**LptDm8**	GDTNDLPVTINADHAKGDYPDDAVFTGSVDIMQGNSRLQADEVQLHQKEAPG<br>QPEPVRTVDALGNVHYDDNQVILKGPKGWANLNTKDSVWYEPEAKVVGKPGS<br>LTADKIETLNNGKETLIEGTNSVVSYNGKTVKIEEKFTIESKLPLDETIQKLSKLL<br>VEGKKPEEAEEILKKEA
**LptDm9**	GDTNDLPVTINADHAKGDYPDDAVFTGSVDIMQGNSRLQADEVQLHQKEAPG<br>QPEPVRTVDALGNVHYDDNQVILKGPKGWANLNTKDWKFWKASIEVVGKPGS<br>ASADVIESKYNGKVVVLYDEKTKKYHVSAFSNLSEDLIKIKEGKAEWVSEEEKE<br>KKIEEVYKKAL
**LptDm10**	GDTNDLPVTINADHAKGDYPDDAVFTGSVDIMQGNSRLQADEVQLHQKEAPG<br>QPEPVRTVDALGNVHYDDNQVILKGPKGWANLNTKDAVFYDASIEVVGEPGSA<br>SADVIEFKYNGKVVVFYNKKTKKYFVTKFEDLPKALIDIKNGTAEWVDEKEKEE<br>VIEKVYKESK
**LptDm11**	GDTNDLPVTINADHAKGDYPDDAVFTGSVDIMQGNSRLQADEVQLHQKEAPG<br>QPEPVRTVDALGNVHYDDNQVILKGPKGWANLNTKDAVFWNPSIKIVGKPGE<br>TSAEKIEFLNNGKTIKLVESDLSELPESVLEELLKEHKNAISIKKETENGKKVVVI<br>EAKTEEDAINLLASLMEKHIELKK
**LptDm12**	GDTNDLPVTINADHAKGDYPDDAVFTGSVDIMQGNSRLQADEVQLHQKEAPG<br>QPEPVRTVDALGNVHYDDNQVILKGPKGWANLNTKDWKFENASIEFVGLPGS<br>GSAKLIKSLDNGQVLLIEEGELSPEFFAAAKAIGIKTLILENPTENALKVLPLAKE<br>AGIKVELV
**LptDm13**	GDTNDLPVTINADHAKGDYPDDAVFTGSVDIMQGNSRLQADEVQLHQKEAPG<br>QPEPVRTVDALGNVHYDDNQVILKGPKGWANLNTKDAKFENASWEAVGKPGS<br>GSADLIYFLANGEIVVLVEKKDGKWYVKVSTFEDLLEGKILVSLELSKEEAEKLL<br>EEMKKLNAEEILKLAKEKKEE
**LptDm14**	GDTNDLPVTINADHAKGDYPDDAVFTGSVDIMQGNSRLQADEVQLHQKEAPG<br>QPEPVRTVDALGNVHYDDNQVILKGPKGWANLNTKDSKWENASWEAVGKPG<br>SGSADLIYFLANGKIVVLLKEEDGKYIIKLSTFEDFLKGETIVELETSKEEAEKLIE<br>EAKTLDADGIIELFKKAKKE

**LptDm15**	GDTNDLPVTINADHAKGDYPDDAVFTGSVDIMQGNSRLQADEVQLHQKEAPG QPEPVRTVDALGNVHYDDNQVILKGPKGWANLNTKDARFENASYEAVGKPGS GSADKIYFLANGKIVVLVKKEDGKWYVELSTFEDLLEGKKLVSLELSKEEAEKLL EEAKKLDAEEIIELFKKKKEE
**LptDm16**	GDTNDLPVTINADHAKGDYPDDAVFTGSVDIMQGNSRLQADEVQLHQKEAPG QPEPVRTVDALGNVHYDDNQVILKGPKGWANLNTKDAVFYDATIETVGAPGSA SAKKIEFKDNGKTLVLDNATFKSYEEAKSLVEYFIKKMGVKTIIVNNATGAITEE LKKELEKLAKELGAKLTITLK
**LptDm17**	GDTNDLPVTINADHAKGDYPDDAVFTGSVDIMQGNSRLQADEVQLHQKEAPG QPEPVRTVDALGNVHYDDNQVILKGPKGWANLNTKDWVFYDASIETVGAPGS ASAKVIKSLDNGKTLEFDDATFDSYENAEALIEHAIKTMGVKTITINNATGAITA EKATKLKAKAAELGATLTITLK
**LptDm18**	GDTNDLPVTINADHAKGDYPDDAVFTGSVDIMQGNSRLQADEVQLHQKEAPG QPEPVRTVDALGNVHYDDNQVILKGPKGWANLNTKDWTFEDASIELVGRPGS GSAKVISSLDNGKKVSFEGPDGTKVTLTTEDAFKILEENDGADAETIVKAAKEYV EKK
**LptDm19**	GDTNDLPVTINADHAKGDYPDDAVFTGSVDIMQGNSRLQADEVQLHQKEAPG QPEPVRTVDALGNVHYDDNQVILKGPKGWANLNTKDAVFYEVSFEAVGRPGSG SADVFKFLDNGEKVEVEIGDKKALADKDKIIEYDSLEALKALAAAAKALGWEKT EKLINEKIKELE
**LptDm20**	GDTNDLPVTINADHAKGDYPDDAVFTGSVDIMQGNSRLQADEVQLHQKEAPG QPEPVRTVDALGNVHYDDNQVILKGPKGWANLNTKDSVWYDVSAEVVGEEGS FSAEKFETRNNGSLLVAENANTEDFIKFAKSLAKVTGKSILKFVKATKDGKTLNI VGISTEEDKEATLAAIEKLVKEKEKEGWKVEEIK
**FL-LptDm**	GDTNDLPVTINADHAKGDYPDDAVFTGSVDIMQGNSRLQADEVQLHQKEAPG QPEPVRTVDALGNVHYDDNQVILKGPKGWANLNTKDWVFYDASIETVGAPGS ASAKVIKSLDNGKTLEFDDATFDSYENAEALIEHAIKTMGVKTITINNATGAITA EKATKLKAKAAELGATLTITLKC-FL(Alexa Fluor 647)
**LptDmAvi**	GDTNDLPVTINADHAKGDYPDDAVFTGSVDIMQGNSRLQADEVQLHQKEAPG QPEPVRTVDALGNVHYDDNQVILKGPKGWANLNTKDWVFYDASIETVGAPGS ASAKVIKSLDNGKTLEFDDATFDSYENAEALIEHAIKTMGVKTITINNATGAITA EKATKLKAKAAELGATLTITLKGSGLNDIFEAQKIEWHE
**mLptA**	GVTGDTDQPLNITADSSVEDKAGNVVTFTGNVIVTQGTIKINADKVVVTAPGGE QGKEVIDGYGKPATFYQMQDNGKPVEGHASQMHYELAKDFVVLTGNAYLQQV DSNIKGDKITYLVKEQKMQAFSDKGKRVTTVLVPSQLQDKNNKGQTPAQKKGN
**mLptAm**	GVTGDTDQPLNITADSSVEDKAGNVVTFTGNVIVTQGTIKINADKVVVTAPGGE QGKEVIDGYGKPATFYQMQDNGKPVEGHASQMHYELAKDFVVLTGNAYLQQV DSNIKGDKITYLVKEQKMQAFSDKGKR

Table S1: Amino acid sequence of all constructs

BUFFER	Protein	Ligand	Kd (nM)	FL ligand conc. (nM)	Competitor conc. (nM)	KD_1 (NM)
TWEEN buffer	mLptA2	FL-149	4190 ± 110	10	-	-
	LptAm	FL-149	6.44 ± 0.24	10	-	-
	Thanatin	LptAm	0.209 ± 0.022	10	24	6.44
	mLptA2	LptAm	139 ± 4	10	50	6.44
	LptDm1	FL-145	458 ± 21	10	-	-
	LptDm4	FL-145	366 ± 19	10	-	-
	LptDm9	FL-145	403 ± 11	10	-	-
	LptDm12	FL-145	953 ± 35	10	-	-
	LptDm17	FL-145	332 ± 13	10	-	-
	LptDm20	FL-145	353 ± 9	10	-	-
	LptDm17-FL	mLptA2	986± 124	5	-	-
	LptDm17-FL	mAm	29800 ± 15700	5	-	-
	LptDm17	FL-145	511 ± 20	10	-	-
	Thanatin	LptDm17	122 ± 24	10	800	511
	peptide1	LptDm17	173 ± 22	10	800	511
	**7**	LptDm17	35.6 ± 6.6	10	800	511
LDAO buffer	LptDm17	FL-145	648 ± 49	10	-	-
	Thanatin	LptDm17	32.4 ± 16.2	10	800	648
	peptide1	LptDm17	123 ± 30	10	800	648
	**7**	LptDm17	30.8 ± 15.6	10	800	648
Screening buffer	LptDm17	FL-145	131 ± 6	10	-	-
	Thanatin	LptDm17	33.0 ± 4.2	10	1100	131
	peptide1	LptDm17	57.9 ± 8.3	10	1100	131
	**7**	LptDm17	4.09 ± 1.54	10	1100	131
Screening buffer + 0.05% BSA	LptDm17	FL-145	203 ± 11	10	-	-
	Thanatin	LptDm17	48.4 ± 7.4	10	1100	203
	peptide1	LptDm17	70.7 ± 12.0	10	1100	203
	**7**	LptDm17	9.30 ± 2.43	10	1100	203

Table S2: Binding affinities of LPT proteins' candidates. Kds as determined by FP are depicted together with experimental buffers of the measurements. For the raw data see Fig. S8. Note that Kd of thanatin binding to the wild-type LptD was determined in LDAO buffer.

	Peptides	-10log$_{10}$P	Δ m [ppm]	m/z	charges
1	GSKKPVPII**Y**CNRRTGKCQR**Y**	90.86	-0.3	517.0777	+5
2	GSKKPVPII**P**CNRRTGKCQR**P**	81.85	1.3	490.6743	+5
3	GSKKPVPII**L/I**CNRRTGKCQR**L/I**	79.66	1.9	497.0871	+5
4	GSKKPVPII**F**CNRRTGKCQR**G**	75.37	1.4	492.6712	+5
5	GSKKPVPII**A**CNRRTGKCQR**F**	67.75	1.6	413.0632	+6
6	GSKKPVPII**Y**CNRRTGKCQR**S**	67.45	0.7	501.8719	+5
thanatin	GSKKPVPII**Y**CNRRTGKCQR**M**	53.60	0.4	510.6735	+5

Table S3: Top scoring peptides from the library screening. Masses of peptides from pulldown experiments as determined by *LC-MS/MS*. Matching scores are specified as -10log$_{10}$P, describing the possibility that the sequence matches the expectation. Mass uncertainties are specified as mass Δm. The measured monoisotopic precursor signals are shown as m/z and the respective charge states. Thanatin is shown as a reference peptide.

LptD(25-223)
LptD(25-174)
LptD(44-160)
LptD(44-144)
LptD(51-91)
LptD(51-78)
mLptA-LptD(44-144)

Table S4: List of LptD constructs that were unfolded and unable to bind thanatin or mLptA

	**LptDm:Thanatin**
**PDB code**	9T3O
**BMRB code**	35025
**NOE distance restraints**	
Total	4052
Intraresidue, i-j=0	810
Sequential, i-j=1	1113
Short-range, i-j=<1	1923
Medium-range, 1<i-j<5	540
Long-range, i-j ≥ 5	1589
Intermolecular	120
Torsion angle constraints	298
**Structure statistics (20 conformers)**	
CYANA target function value ($Å^2$)	3.34 ± 0.58
**Satisfaction of Experimental Constraints**	
Distance constraint violation	
Number > 0.2 Å	4 ± 1
Maximum (Å)	0.26 ± 0.04
Van der Waals violations	
Number > 0.2 Å	7 ± 1
Maximum (Å)	0.35
Torsion angle constraint violations	
Number > 5°	2 ± 1
Maximum (deg)	6.17 ± 0.44
PROCHECK Ramachandran plot analysis	
Residues in favored regions (%)	83.5 %
Residues in additional allowed regions (%)	16.5 %
Residues in generously allowed regions (%)	0.0 %
Residues in disallowed regions (%)	0.0 %
**RMSD to the average coordinates (Å)**	
Backbone atoms	0.62 ± 0.13
Heavy atoms	0.82 ± 0.10

Table S5: Statistics from the NMR structure calculation

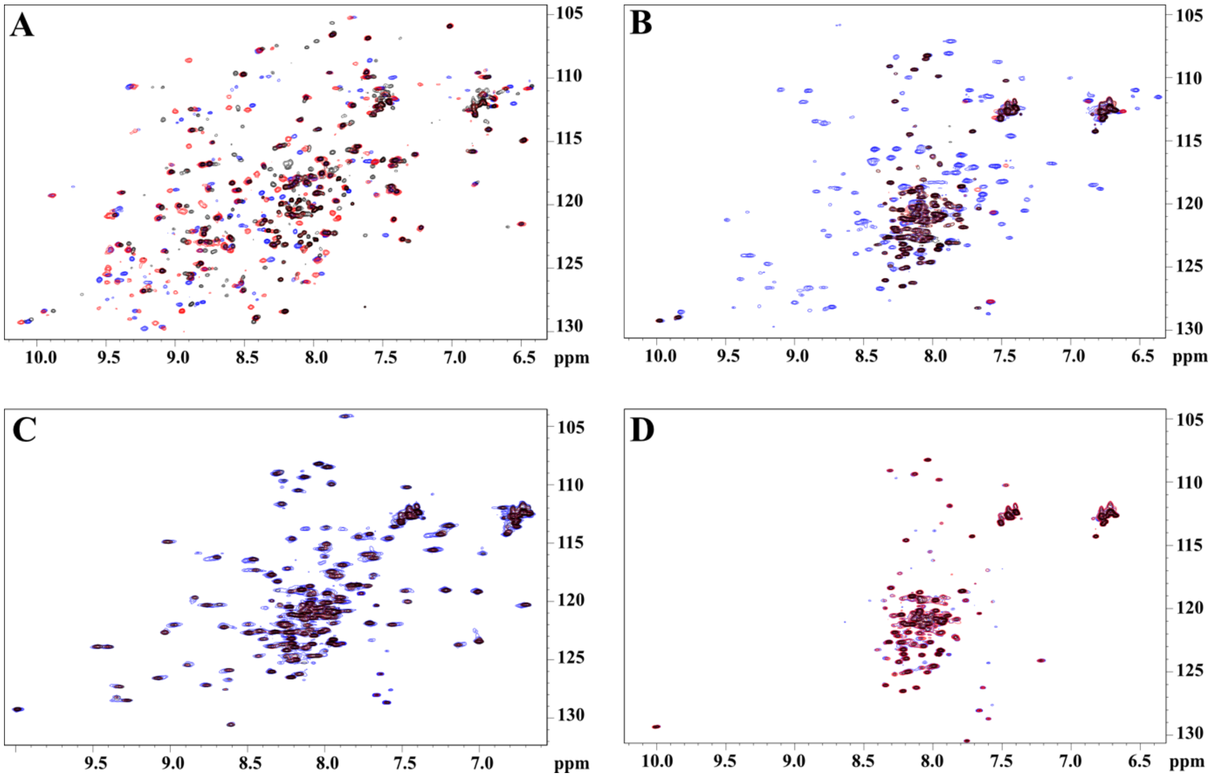

Figure S1: Characterization of folding and thanatin-binding by NMR. Overlay of [^{15}N,^{1}H]-HSQC spectra of designed LptD proteins in their apo form (black contours), their thanatin-complex (blue contours) and when bound to LptA (red contours). **(A)** shows a case when all species are folded and LptD binds to both thanatin and LptA, **(B)** gives an example when the apo species is unfolded but the thanatin complex is folded and LptA is not bound, and **(C)** when the apo protein is folded but does not bind to thanatin or LptA, and **(D)** when LptD is unfolded in its apo state and when mixed with LptA or thanatin.

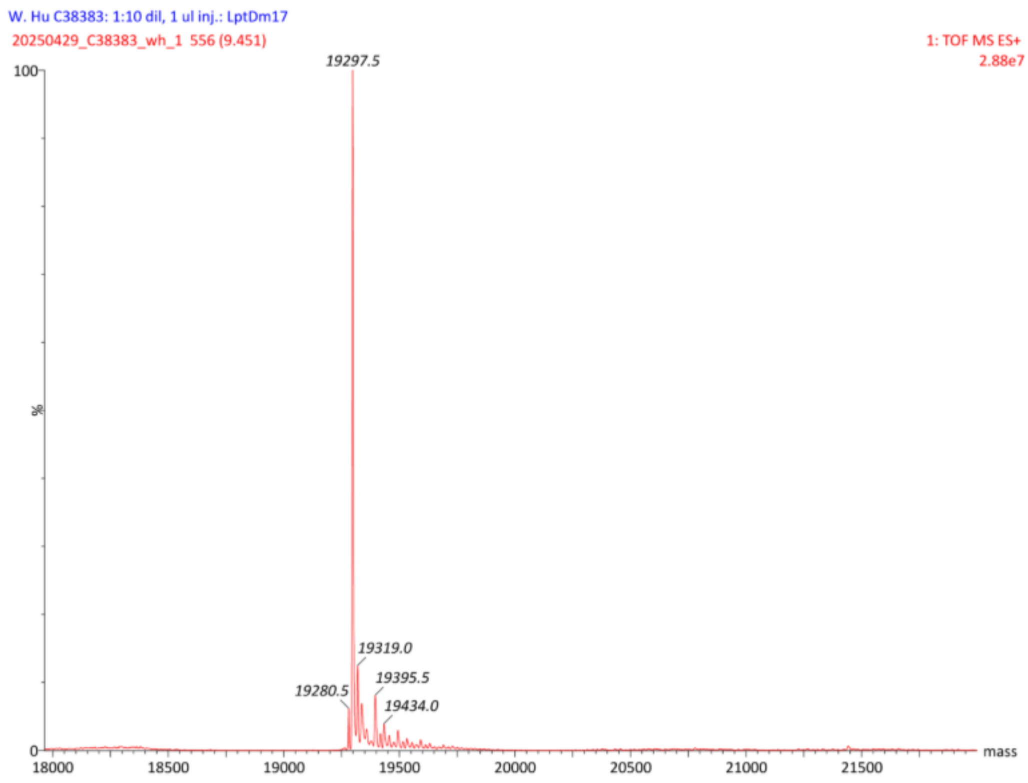

Figure S2: The LC-MS spectrum of LptDm17. Theoretical mass is 19296.48 Da.

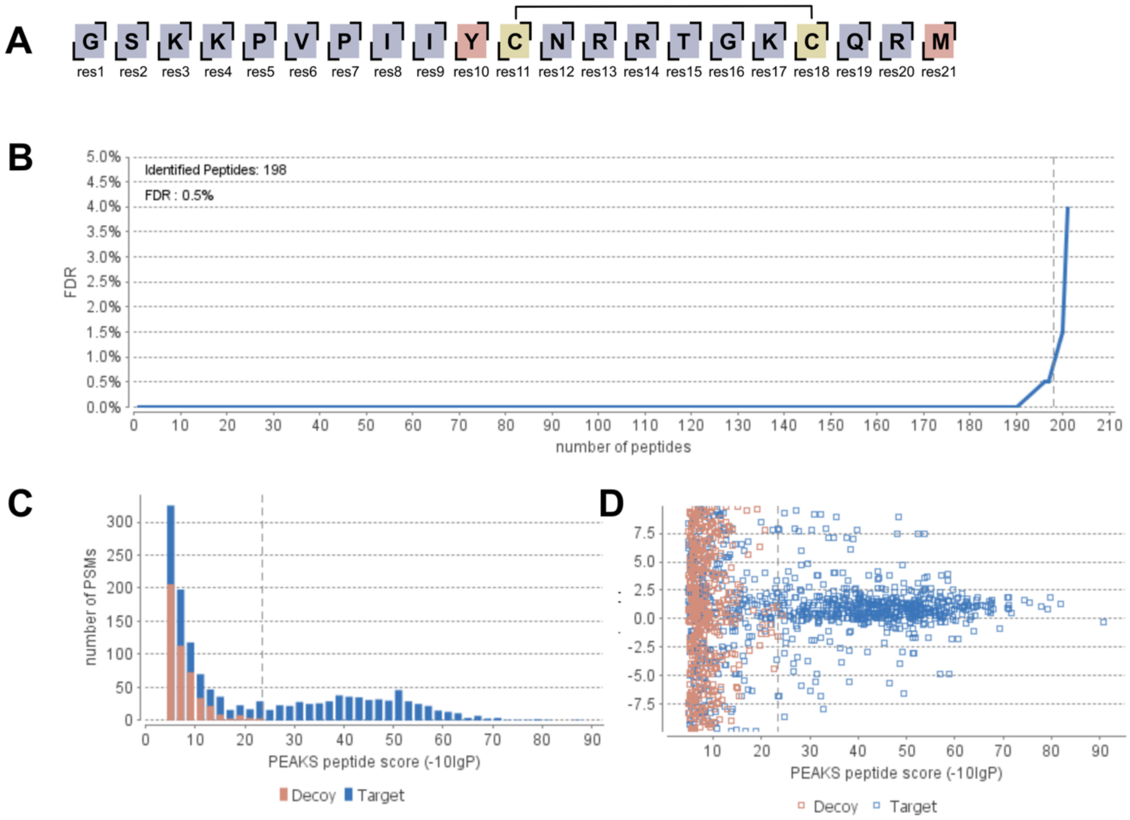

Figure S3: The design of the peptide library and LC-MS/MS results of the pull-down. (A) Design of the peptide library. Mutated residues are highlighted in red, and a disulfide bond, as present in thanatin, is introduced between two cysteines shown in yellow. **(B)** Number of peptides identified from the library at different false-discovery-rate (FDR) thresholds, where the FDR serves as a proxy for identification of uncertainty. **(C)** The number of Peptide-Spectrum Match (PSMs) distribution versus the Matching Scores. **(D)** Scatterplot of mass matching scores versus precursor mass error with the decoy data base as the control.

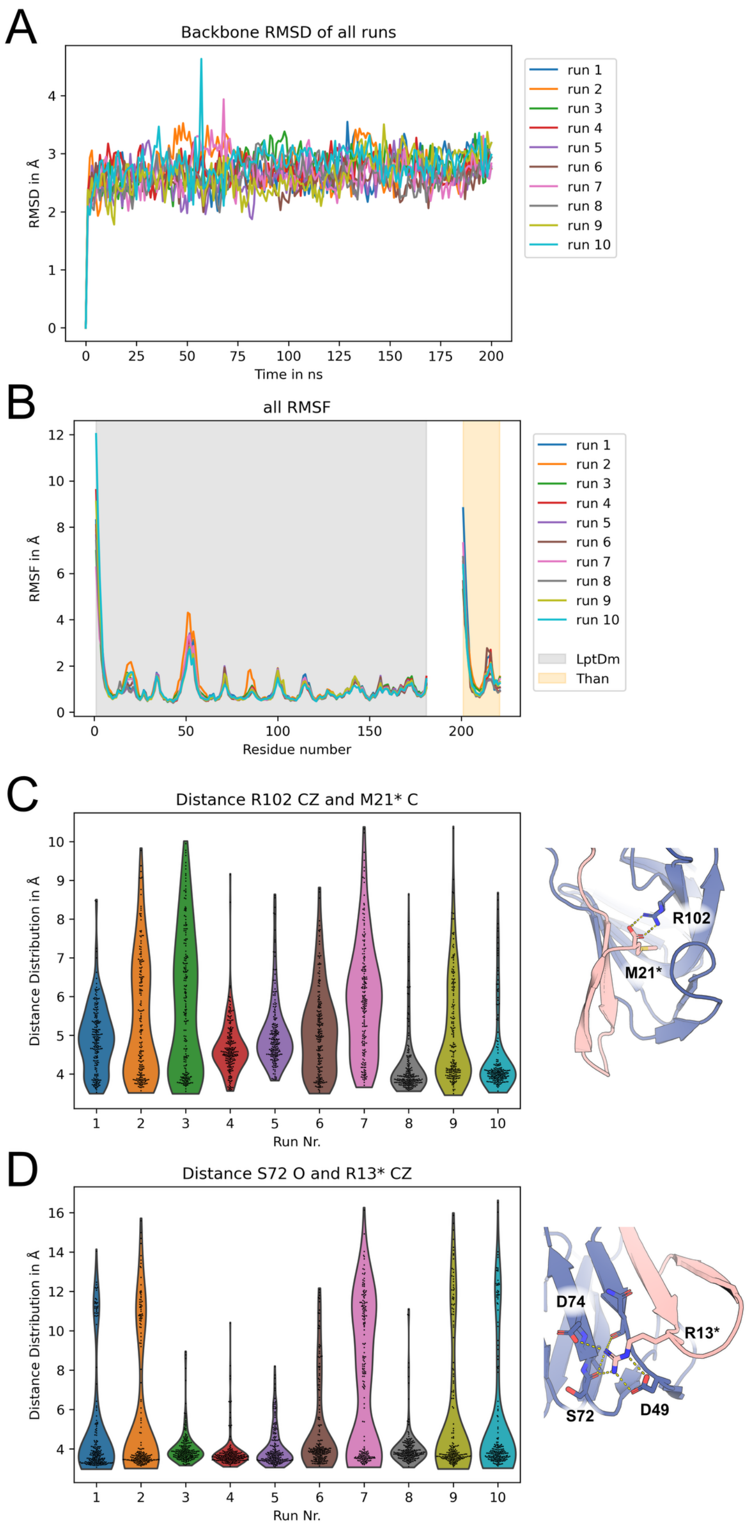

Figure S4: MD results of the lowest energy conformer of the LptDm NMR structure. A) Backbone RMDS of the individual runs. Each run was calculated for 200 ns. **B)** RMSF of the Cα atoms of the individual runs. **C)** Distance distribution of Cς(R102) to C'(M21*) in the individual runs. **D)** Distance distribution of O of S72 to Cς(R13*) in the individual runs.

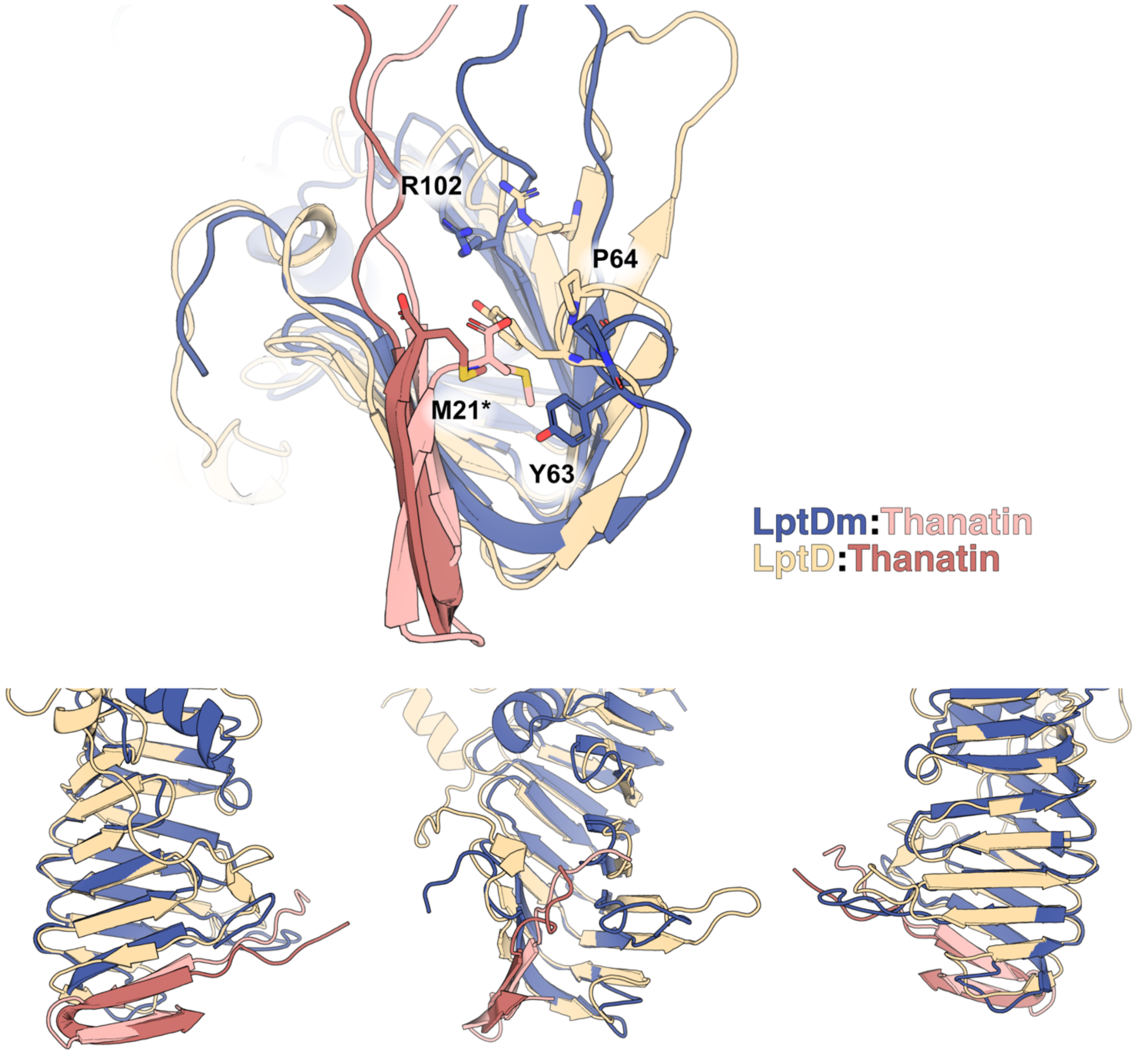

Figure S5: Structure comparison of the cryo-EM structure of the *E. coli* LptDEM:thanatin complex with the LptDm:thanatin NMR structure

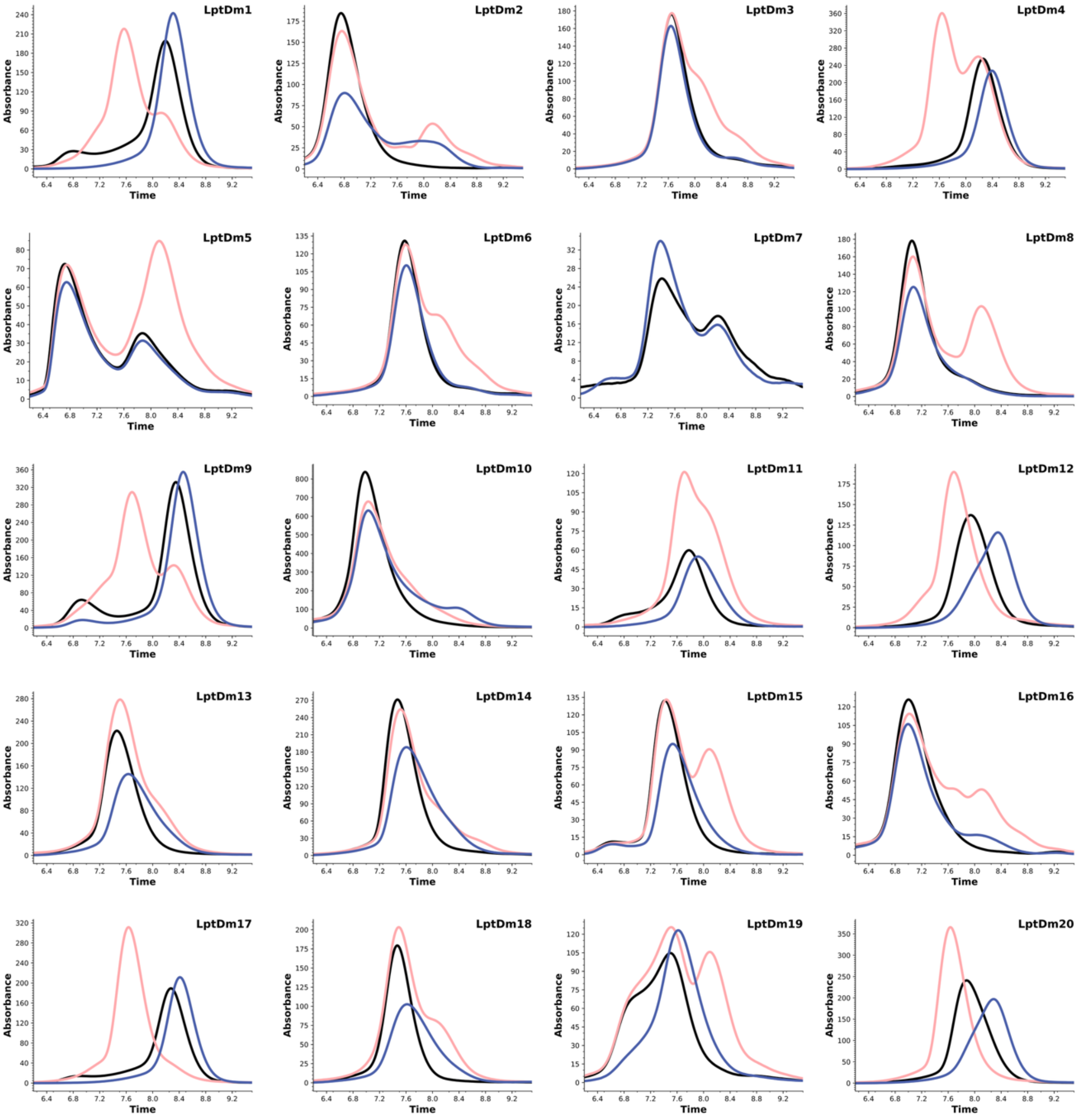

Figure S6: SEC plots of all apo-state LptDm variants (black), LptDm:thanatin complexes (blue), and LptDm:mLptA complexes (red).

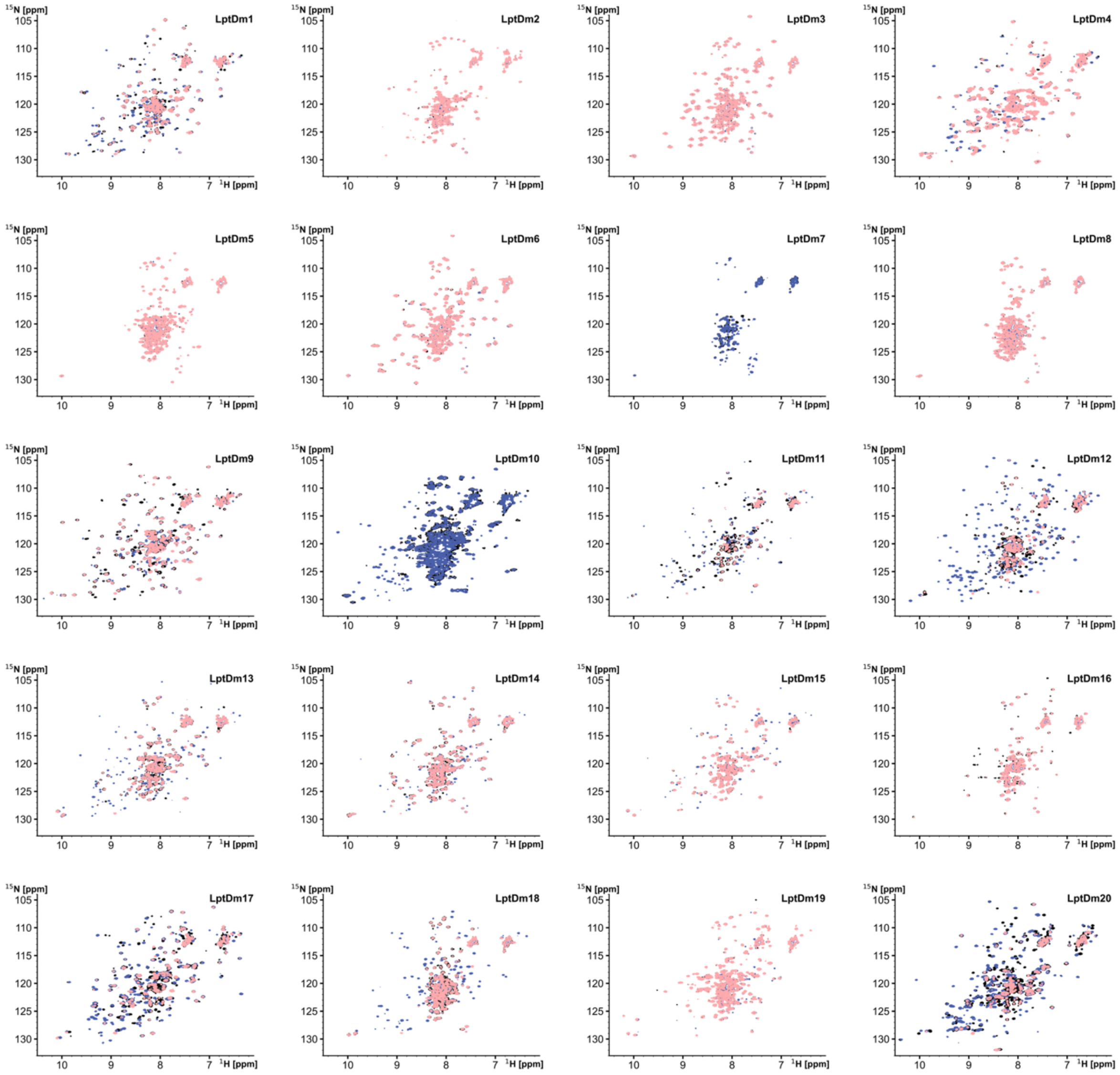

Figure S7: Overlays of [15N,1H]-HSQC spectra of all apo-state LptDm variants (black), LptDm:thanatin complexes (blue), and LptDm:mLptA complexes (red).

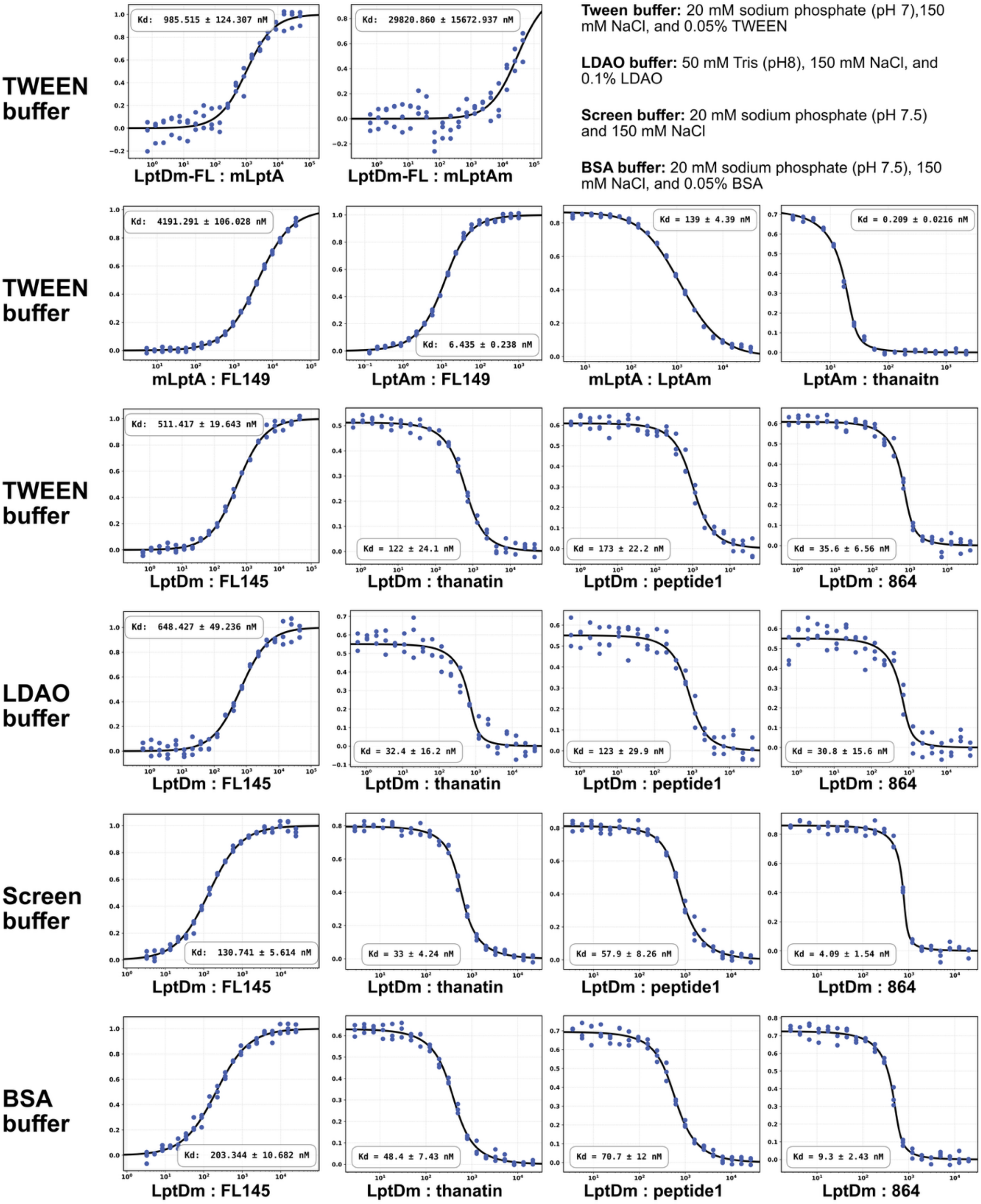

Figure S8: Overview of all FP measurements.

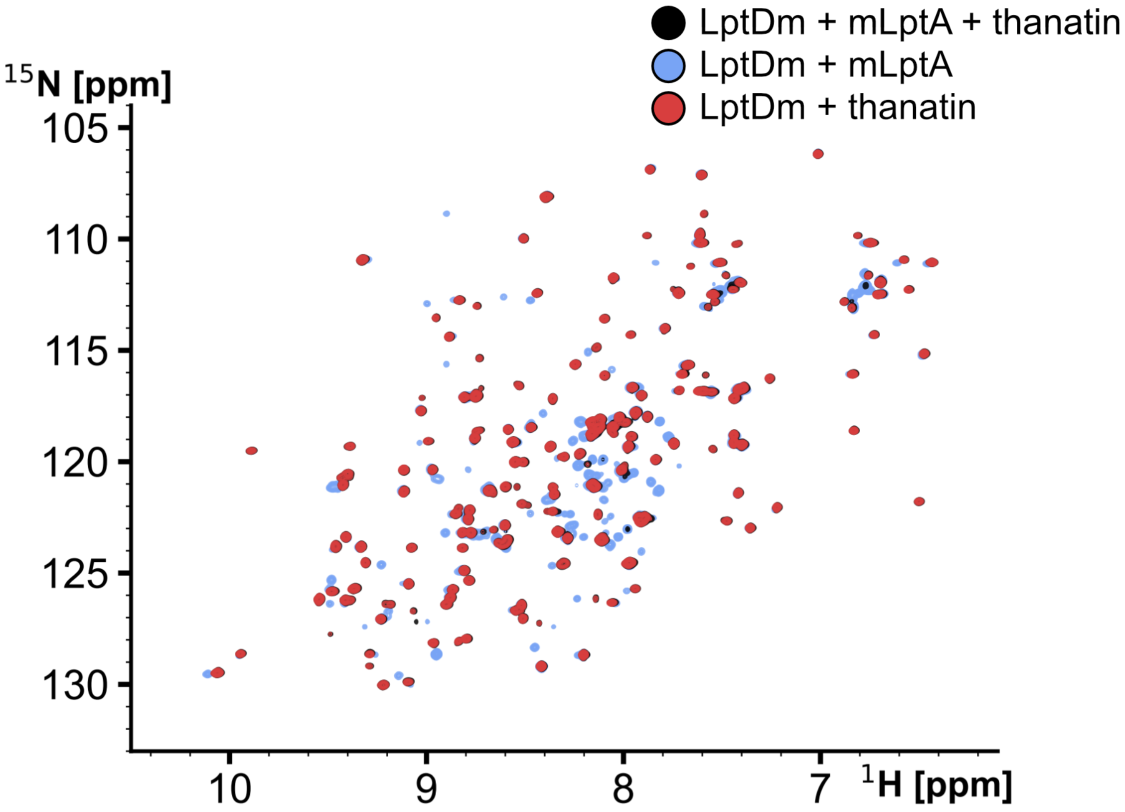

Figure S9: [^{15}N,^{1}H]-HSQC data demonstrating disassembly of the LptDm:mLptA complex upon addition of thanatin. Contours of the LptDm:mLptA complex are colored in blue, LptD:thanatin in red and LptDm:mLptA:thanatin (1:1:2) in black. All peaks in the LptD:thanatin complex perfectly superimpose with the peaks in the LptDm:mLptA:thanatin mixture, and many from the latter deviate from those of the LptDm:mLptA complex.

construct name	Open Reading Frame (ORF)
**LptDm01**	ATGTCTGGTTCTCATCATCATCATCATCATAGCAGCGGCATCGAAGGCCGCGGCCGCCAGTACAAA<br>CTGATCCTGAACGGTAAAACCCTGAAAGGTGAAACCACCACCGAAGCTGTTGACGCTGCTACCGCG<br>GAAAAAGTTTTCAAACAGTACGCTAACGACAACGGTGTTGACGGTGAATGGACCTACGACGACGCT<br>ACCAAAACCTTCACCGTTACCGAAAGCAGCGGCGAAAACCTGTACTTCCAGGGCGATACAAATGAC<br>TTACCTGTGACGATTAATGCTGACCATGCGAAGGGGGACTATCCGGATGACGCAGTGTTCACCGGT<br>TCAGTAGACATCATGCAAGGCAATTCTCGATTACAGGCAGATGAAGTACAACTTCATCAAAAAGAAG<br>CCCCTGGGCAACCGGAGCCTGTGCGGACCGTAGATGCCCTCGGGAACGTACACTATGACGACAAC<br>CAAGTGATCCTTAAAGGTCCAAAAGGCTGGGCCAATTTAAATACTAAAGATTGGAAATTTGAAGACG<br>CTTCCATTGAGTTTGTGGGCATGCCGGGCTCGGCTAGCGGAAAATTGATAGAGTATAAAAATGGTAC<br>CATTACTATCTACATCGATGAAAATTCCAAAATAACGGAAGAGTTTATTGAAGCGGCGACAAAGCTTG<br>GGTACAAAGTAGAAGTAAAAGGTGATGAGAAAGTAAAAGAGAAGGTGGATGAACTTAAAGAAATAAT<br>TAAAAAAGTCGAAGCGAAATAA
**LptDm02**	ATGTCTGGTTCTCATCATCATCATCATCATAGCAGCGGCATCGAAGGCCGCGGCCGCCAGTACAAA<br>CTGATCCTGAACGGTAAAACCCTGAAAGGTGAAACCACCACCGAAGCTGTTGACGCTGCTACCGCG<br>GAAAAAGTTTTCAAACAGTACGCTAACGACAACGGTGTTGACGGTGAATGGACCTACGACGACGCT<br>ACCAAAACCTTCACCGTTACCGAAAGCAGCGGCGAAAACCTGTACTTCCAGGGCGACACCAACGAT<br>CTGCCCGTAACCATAAATGCGGACCATGCTAAAGGCGATTATCCAGATGACGCAGTTTTTACAGGCA<br>GCGTTGATATCATGCAAGGAAATTCGCGCCTGCAAGCTGATGAAGTGCAGCTGCATCAGAAAGAGG<br>CTCCGGGTCAGCCAGAACCGGTTCGAACGGTCGATGCCCTGGGCAATGTCCACTACGATGACAAC<br>CAGGTCATCCTTAAAGGGCCTAAAGGATGGGCAAACTTAAATACGAAAGATTCAGTGTGGTACAATA<br>GTTCTGTAGAAAGCGTCGGTGCGCCTGGTTCCTTCTCAGCAGAGATCATTCTTAGTCTGTTAAACGG<br>GAAGAGTTTGGTCGCGGTTGATATTAGCGAAGAGGACAAACCTGGTTTCAAAGAAGTTGCTAAGAAC<br>CTGGGCCTGAAAGTTTTTCTTGAAACAGAAGTGAACGGAAAAAAGACACTGATAGCGTCGAAGGAC<br>GAAGAAGACGTGCCAAAGGCGTTGGAAAAAGCAGAAAAGATAGTGAAAGAGGTCGAAAAGGAATAA
**LptDm03**	ATGTCTGGTTCTCATCATCATCATCATCATAGCAGCGGCATCGAAGGCCGCGGCCGCCAGTACAAA<br>CTGATCCTGAACGGTAAAACCCTGAAAGGTGAAACCACCACCGAAGCTGTTGACGCTGCTACCGCG<br>GAAAAAGTTTTCAAACAGTACGCTAACGACAACGGTGTTGACGGTGAATGGACCTACGACGACGCT<br>ACCAAAACCTTCACCGTTACCGAAAGCAGCGGCGAAAACCTGTACTTCCAGGGTGACACTAATGAC<br>CTTCCTGTCACCATTAATGCTGATCATGCGAAAGGAGATTACCCGGATGATGCTGTTTTTACGGGTT<br>CCGTCGATATAATGCAGGGTAACAGCCGCTTGCAAGCAGATGAGGTACAGTTGCATCAAAAGGAAG<br>CTCCGGGACAACCGGAGCCGGTGCGTACTGTTGATGCTCTGGGTAATGTGCATTATGACGATAATC<br>AAGTTATCTTAAAAGGTCCCAAAGGCTGGGCTAATCTGAACACTAAAGACTCCGTATGGTTTGACCC<br>ATCGGCAGAAATGGTTGGTTTACCTGGATCAGGGAGTGCGAAAAAAATTGAGTTTTTGGACAATGGT<br>AAGCTGGTGAAACTGGTGATCGATAAAGAACATTACGAGAAAGTTAAAGAGGAACTTTTGAAAACTG<br>TCGATGCCAAAGTTGAAGAACTCCCGGATGGCTCGGTTGCTATTACGACAAGTGGTGATCCAGAGA<br>CGGTCAGCAAAACTTTGTCAATCCTTAAGTAA
**LptDm04**	ATGTCTGGTTCTCATCATCATCATCATCATAGCAGCGGCATCGAAGGCCGCGGCCGCCAGTACAAA<br>CTGATCCTGAACGGTAAAACCCTGAAAGGTGAAACCACCACCGAAGCTGTTGACGCTGCTACCGCG<br>GAAAAAGTTTTCAAACAGTACGCTAACGACAACGGTGTTGACGGTGAATGGACCTACGACGACGCT<br>ACCAAAACCTTCACCGTTACCGAAAGCAGCGGCGAAAACCTGTACTTCCAGGGCGATACCAATGAT<br>CTGCCTGTTACCATCAATGCTGATCACGCAAAAGGTGATTATCCAGACGATGCCGTTTTCACAGGTA<br>GCGTGGACATAATGCAGGGTAATTCCAGACTGCAGGCTGATGAAGTTCAGCTTCACCAAAAGGAAG<br>CCCCTGGCCAACCGGAGCCGGTTAGAACGGTGGATGCATTAGGCAACGTACACTACGATGATAACC<br>AGGTTATTTTAAAAGGTCCTAAAGGATGGGCCAATCTCAATACGAAAGATGCGACCTTTTGGGAGGC<br>TAGCATGGAGTTCGTAGGGGCCCCAGGCGCCTTCACCGCGGACGAAATTAAATTCCTTGATAATGG<br>CAAAATAGTGGAATTAATACTCAATGGTAAAACGGCGAAATTTCTGTTTCCGGAACTGCTGAAGACAT<br>CAAAAGAGGCGAAAGAAAAAGGCTGGAGCAAAGAAGAACTTATTGAGGCATTTAAAAAAACCCTCCT<br>GCCAGGTAGTGACGCTGAAGTCCTGGAGGAGGCCATGAAGAAAGCCTAA
**LptDm05**	ATGTCTGGTTCTCATCATCATCATCATCATAGCAGCGGCATCGAAGGCCGCGGCCGCCAGTACAAA<br>CTGATCCTGAACGGTAAAACCCTGAAAGGTGAAACCACCACCGAAGCTGTTGACGCTGCTACCGCG<br>GAAAAAGTTTTCAAACAGTACGCTAACGACAACGGTGTTGACGGTGAATGGACCTACGACGACGCT<br>ACCAAAACCTTCACCGTTACCGAAAGCAGCGGCGAAAACCTGTACTTCCAGGGCGATACAAATGAT<br>CTGCCGGTTACAATTAACGCAGATCATGCCAAAGGAGACTACCCGGATGACGCAGTGTTTACGGGC<br>AGCGTGGATATCATGCAGGGCAATAGCAGACTGCAAGCAGATGAGGTCCAATTGCATCAGAAGGAA<br>GCCCCGGGGCAACCAGAACCCGTTCGTACCGTGGACGCGTTAGGTAATGTCCATTATGATGATAAC<br>CAAGTTATTCTCAAAGGACCTAAAGGTTGGGCTAACCTTAACACCAAGGACGCACGTTTCGAAGATG<br>CGAGTATGGAATTCGTCGGCGTTCCCGGGTCCGCATCAGCAACCCTTATAGAAAAAAAAGATGGTG<br>CATACTTCTTTAAAGATCCCACGTTTAAAGATTTGTCAGAGGAACTGAAAGAACTGGTGCTGGAAGC

	AGACGAACTTTTGAAAAAAGGCGATAAAAAAGAGGCACTGAAGAAAGCATATGAATTTGAAAAACTT<br>GTTGCAGAATTGTTGGGGAACAAAGAAGAGGTGGAGAAACTGGAAAAAAAGCTCAAGGAGGTTTAA
**LptDm06**	ATGTCTGGTTCTCATCATCATCATCATCATAGCAGCGGCATCGAAGGCCGCGGCCGCCAGTACAAA<br>CTGATCCTGAACGGTAAAACCCTGAAAGGTGAAACCACCACCGAAGCTGTTGACGCTGCTACCGCG<br>GAAAAAGTTTTCAAACAGTACGCTAACGACAACGGTGTTGACGGTGAATGGACCTACGACGACGCT<br>ACCAAAACCTTCACCGTTACCGAAAGCAGCGGCGAAAACCTGTACTTCCAGGGTGATACAAATGATC<br>TTCCCGTAACAATTAACGCGGATCACGCCAAGGGAGACTATCCTGATGATGCTGTTTTCACAGGAAG<br>CGTTGATATTATGCAGGGAAATTCTCGTTTGCAGGCAGATGAAGTACAATTGCACCAGAAGGAGGC<br>CCCGGGCCAGCCTGAACCGGTGCGTACCGTTGATGCACTCGGTAATGTACATTATGACGACAACCA<br>GGTTATCCTCAAAGGCCCCAAAGGCTGGGCCAACCTCAATACCAAAGATGCCGTGTTTGAGGATGC<br>AAGCGGCGAAGTTGTAGGTCTGCCAGGAAGCGCTTCCGCCAAAAAAATCGAGTTTAAAAGTAATGG<br>CAATATTATCGTCTTTTACGATGTTGAAGATGTGGAGGAAGCCATAAAGTTCGCAGAGTCTTACTTTA<br>AGGCACAGGGAAAAGAGGTCGATCTTATTATTGTGAAGGGCAAAAATGTGATAGCTTCAACAGAAGA<br>GGCAAAAGAGGAAGCTAAGAAAATTGCAGAGAAAATTTAA
**LptDm07**	ATGTCTGGTTCTCATCATCATCATCATCATAGCAGCGGCATCGAAGGCCGCGGCCGCCAGTACAAA<br>CTGATCCTGAACGGTAAAACCCTGAAAGGTGAAACCACCACCGAAGCTGTTGACGCTGCTACCGCG<br>GAAAAAGTTTTCAAACAGTACGCTAACGACAACGGTGTTGACGGTGAATGGACCTACGACGACGCT<br>ACCAAAACCTTCACCGTTACCGAAAGCAGCGGCGAAAACCTGTACTTCCAGGGAGACACTAATGAT<br>CTTCCAGTTACTATCAATGCCGATCACGCTAAGGGCGATTATCCCGATGACGCCGTTTTTACGGGAA<br>GCGTGGACATCATGCAGGGAAATAGCCGTCTGCAAGCCGATGAAGTCCAGCTGCATCAGAAAGAAG<br>CTCCGGGTCAACCTGAACCAGTTCGTACCGTGGATGCGCTGGGCAACGTACATTACGATGATAATC<br>AGGTAATCCTTAAAGGTCCGAAAGGTTGGGCGAATTTGAACACAAAGGATGGGGTTTTTTACGACGC<br>ATCAGCAGAAAACGTCGGCGCACCAGGTTCCGCTTCAGCGAAAAAAATAGAATTTCTGAACAACGG<br>AAAAATCCTGGTTTTTTACGATGCAACCTCATCTGAGGAAGATGTCAAGACTCTCGCCGAAAATTATC<br>TTAAACAACTGGGTGAGGAAGCGGTGTTCGAATCGATTGATGGCAATTTTATAGCAGCAGCAAAATC<br>TAAAAAAGCTGAGCTTGAAAAGCTCGCGGCCGAGCTGAAATAA
**LptDm08**	ATGTCTGGTTCTCATCATCATCATCATCATAGCAGCGGCATCGAAGGCCGCGGCCGCCAGTACAAA<br>CTGATCCTGAACGGTAAAACCCTGAAAGGTGAAACCACCACCGAAGCTGTTGACGCTGCTACCGCG<br>GAAAAAGTTTTCAAACAGTACGCTAACGACAACGGTGTTGACGGTGAATGGACCTACGACGACGCT<br>ACCAAAACCTTCACCGTTACCGAAAGCAGCGGCGAAAACCTGTACTTCCAGGGAGATACTAACGAC<br>CTGCCGGTCACAATAAATGCGGATCATGCAAAAGGCGATTACCCGGATGATGCGGTGTTTACTGGC<br>TCGGTGGATATCATGCAGGGAAATTCACGCTTACAGGCTGATGAAGTTCAACTGCATCAGAAAGAAG<br>CCCCTGGCCAGCCTGAACCGGTTCGCACAGTGGACGCTCTGGGAAACGTTCATTATGATGATAATC<br>AAGTCATCCTTAAAGGTCCTAAAGGCTGGGCAAACCTTAACACTAAAGACTCGGTGTGGTATGAACC<br>AGAAGCGAAAGTTGTTGGAAAACCAGGAAGCTTAACTGCTGATAAAATAGAAACGCTCAATAATGGT<br>AAAGAGACTCTGATCGAAGGCACAAACTCGGTCGTTAGCTACAACGGTAAAACAGTTAAAATAGAAG<br>AAAAATTTACTATTGAGTCAAAACTGCCGTTAGATGAGACAATTCAAAAACTGAGCAAGTTGCTGGTA<br>GAAGGTAAGAAGCCTGAAGAGGCAGAAGAGATCCTCAAAAAAGAAGCATAA
**LptDm09**	ATGTCTGGTTCTCATCATCATCATCATCATAGCAGCGGCATCGAAGGCCGCGGCCGCCAGTACAAA<br>CTGATCCTGAACGGTAAAACCCTGAAAGGTGAAACCACCACCGAAGCTGTTGACGCTGCTACCGCG<br>GAAAAAGTTTTCAAACAGTACGCTAACGACAACGGTGTTGACGGTGAATGGACCTACGACGACGCT<br>ACCAAAACCTTCACCGTTACCGAAAGCAGCGGCGAAAACCTGTACTTCCAGGGTGATACCAATGAC<br>CTTCCTGTAACGATTAACGCGGATCATGCAAAAGGAGATTACCCGGATGATGCGGTTTTTACCGGTA<br>GTGTAGACATAATGCAGGGTAACTCACGATTACAGGCAGATGAAGTCCAGCTGCATCAGAAAGAAG<br>CCCCTGGCCAGCCGGAACCGGTTCGGACAGTGGATGCACTGGGGAACGTGCATTATGACGATAAT<br>CAAGTGATCTTAAAAGGCCCAAAGGGATGGGCTAATCTTAACACAAAAGATTGGAAGTTTTGGAAAG<br>CTAGCATTGAGGTTGTAGGGAAACCAGGTTCTGCTTCTGCAGATGTAATCGAATCAAAATATAACGG<br>CAAAGTTGTGGTCCTGTATGATGAAAAAACCAAAAAATATCATGTTAGTGCATTTAGTAATTTGTCCG<br>AAGACCTGATTAAAATCAAGGAGGGTAAAGCTGAGTGGGTGTCTGAGGAAGAAAAAGAGAAAAAAA<br>TAGAGGAAGTCTACAAGAAGGCTTTGTAA
**LptDm10**	ATGTCTGGTTCTCATCATCATCATCATCATAGCAGCGGCATCGAAGGCCGCGGCCGCCAGTACAAA<br>CTGATCCTGAACGGTAAAACCCTGAAAGGTGAAACCACCACCGAAGCTGTTGACGCTGCTACCGCG<br>GAAAAAGTTTTCAAACAGTACGCTAACGACAACGGTGTTGACGGTGAATGGACCTACGACGACGCT<br>ACCAAAACCTTCACCGTTACCGAAAGCAGCGGCGAAAACCTGTACTTCCAGGGGGACACGAACGAT<br>CTGCCAGTTACAATCAACGCCGATCATGCAAAAGGTGACTATCCGGACGATGCTGTTTTTACCGGAA<br>GTGTAGATATAATGCAAGGAAATAGCCGCCTGCAAGCCGATGAGGTGCAGCTTCATCAGAAAGAAG<br>CGCCAGGGCAACCAGAGCCGGTGCGGACCGTCGATGCCCTGGGCAATGTTCATTACGACGATAAT<br>CAAGTTATACTGAAGGGTCCAAAAGGTTGGGCGAATCTGAATACTAAAGATGCGGTTTTTTACGATG<br>CCTCCATCGAAGTCGTAGGTGAGCCGGGCTCTGCATCTGCGGACGTGATTGAGTTTAAGTATAACG

	GTAAAGTAGTCGTTTTTTATAACAAAAAAACTAAGAAGTATTTTGTTACCAAGTTTGAAGATCTGCCCA<br>AAGCCCTTATTGACATTAAAAACGGAACCGCCGAATGGGTGGATGAAAAGGAAAAAGAAGAAGTTAT<br>AGAAAAAGTGTACAAAGAAAGTAAATAA
**LptDm11**	ATGTCTGGTTCTCATCATCATCATCATCATAGCAGCGGCATCGAAGGCCGCGGCCGCCAGTACAAA<br>CTGATCCTGAACGGTAAAACCCTGAAAGGTGAAACCACCACCGAAGCTGTTGACGCTGCTACCGCG<br>GAAAAAGTTTTCAAACAGTACGCTAACGACAACGGTGTTGACGGTGAATGGACCTACGACGACGCT<br>ACCAAAACCTTCACCGTTACCGAAAGCAGCGGCGAAAACCTGTACTTCCAGGGTGATACCAACGAC<br>CTGCCAGTAACAATTAACGCCGATCATGCAAAAGGGGATTACCCGGACGATGCCGTTTTCACCGGT<br>TCTGTTGATATTATGCAGGGAAATTCACGCCTCCAGGCTGATGAGGTACAGCTGCATCAAAAGGAAG<br>CTCCAGGTCAGCCGGAACCCGTCCGCACAGTCGACGCGCTGGGCAATGTGCACTATGATGACAAT<br>CAGGTAATACTCAAAGGGCCGAAAGGTTGGGCGAATCTTAACACAAAGGATGCCGTTTTTTGGAATC<br>CCAGTATCAAAATCGTGGGCAAACCTGGCGAAACTAGCGCAGAAAAAATAGAATTTCTGAATAACGG<br>CAAAACAATCAAACTGGTTGAATCTGATTTGAGCGAGCTGCCGGAATCCGTGCTCGAAGAACTCTTA<br>AAGGAACACAAAAATGCAATCTCAATTAAAAAGGAAACAGAGAATGGTAAGAAGGTCGTAGTAATTG<br>AAGCGAAAACCGAAGAAGATGCGATTAATTTATTAGCCAGTCTGATGGAGAAACATATAGAACTCAA<br>AAAATAA
**LptDm12**	ATGTCTGGTTCTCATCATCATCATCATCATAGCAGCGGCATCGAAGGCCGCGGCCGCCAGTACAAA<br>CTGATCCTGAACGGTAAAACCCTGAAAGGTGAAACCACCACCGAAGCTGTTGACGCTGCTACCGCG<br>GAAAAAGTTTTCAAACAGTACGCTAACGACAACGGTGTTGACGGTGAATGGACCTACGACGACGCT<br>ACCAAAACCTTCACCGTTACCGAAAGCAGCGGCGAAAACCTGTACTTCCAGGGCGACACCAACGAT<br>TTACCAGTGACGATAAATGCAGACCACGCGAAAGGTGACTACCCCGATGATGCAGTTTTTACAGGC<br>AGCGTCGATATCATGCAGGGGAACTCTAGATTGCAGGCGGATGAAGTCCAGCTGCATCAAAAGGAA<br>GCCCCTGGACAGCCGGAGCCAGTCCGTACCGTGGACGCGCTGGGCAACGTCCATTACGACGATAA<br>CCAGGTCATTCTGAAAGGCCCTAAGGGGTGGGCTAATCTGAACACCAAAGATTGGAAATTTGAGAAT<br>GCGTCTATTGAATTTGTTGGGCTGCCGGGATCAGGGTCAGCCAAACTTATTAAAAGTCTCGACAATG<br>GCCAGGTGCTGTTAATCGAGGAGGGTGAGCTGTCCCCGGAATTTTTCGCCGCTGCAAAAGCGATAG<br>GTATTAAAACACTGATTTTAGAGAATCCAACTGAAAATGCTCTGAAAGTGCTGCCGCTGGCTAAGGA<br>AGCAGGAATCAAAGTTGAACTTGTTTAA
**LptDm13**	ATGTCTGGTTCTCATCATCATCATCATCATAGCAGCGGCATCGAAGGCCGCGGCCGCCAGTACAAA<br>CTGATCCTGAACGGTAAAACCCTGAAAGGTGAAACCACCACCGAAGCTGTTGACGCTGCTACCGCG<br>GAAAAAGTTTTCAAACAGTACGCTAACGACAACGGTGTTGACGGTGAATGGACCTACGACGACGCT<br>ACCAAAACCTTCACCGTTACCGAAAGCAGCGGCGAAAACCTGTACTTCCAGGGGGATACTAACGAC<br>CTCCCGGTCACTATTAATGCGGATCATGCAAAAGGGGATTACCCTGACGATGCTGTGTTTACCGGG<br>AGCGTGGACATAATGCAAGGGAACAGCCGCCTGCAAGCGGACGAGGTGCAACTGCATCAGAAGGA<br>GGCACCGGGACAGCCTGAACCTGTCCGCACGGTTGATGCGTTAGGCAACGTGCATTATGATGATAA<br>TCAGGTCATACTGAAAGGTCCCAAAGGTTGGGCTAACCTGAATACCAAAGACGCGAAATTCGAAAAT<br>GCTTCATGGGAGGCAGTGGGCAAACCGGGATCGGGGTCAGCGGATCTGATTTATTTTTTGGCCAAT<br>GGCGAGATAGTTGTCCTCGTTGAAAAAAAAGACGGCAAGTGGTATGTTAAGGTTAGCACATTTGAAG<br>ATTTGTTAGAAGGAAAAATACTaGTGTCCCTCGAGTTGTCTAAAGAAGAAGCCGAAAAACTGCTTGAA<br>GAGATGAAAAAACTCAACGCTGAGGAAATTCTGAAGTTAGCAAAAGAAAAAAAGGAAGAATAA
**LptDm14**	ATGTCTGGTTCTCATCATCATCATCATCATAGCAGCGGCATCGAAGGCCGCGGCCGCCAGTACAAA<br>CTGATCCTGAACGGTAAAACCCTGAAAGGTGAAACCACCACCGAAGCTGTTGACGCTGCTACCGCG<br>GAAAAAGTTTTCAAACAGTACGCTAACGACAACGGTGTTGACGGTGAATGGACCTACGACGACGCT<br>ACCAAAACCTTCACCGTTACCGAAAGCAGCGGCGAAAACCTGTACTTCCAGGGTGATACCAACGAT<br>TTACCGGTGACGATCAATGCTGATCATGCAAAAGGGGACTACCCAGATGATGCCGTCTTTACCGGC<br>TCGGTGGATATAATGCAGGGTAACAGCCGCCTGCAGGCCGATGAGGTCCAGTTACACCAGAAGGA<br>GGCACCAGGCCAGCCGGAGCCGGTTCGCACTGTTGATGCTTTAGGTAATGTTCATTACGACGACAA<br>TCAGGTAATCCTTAAGGGCCCCAAGGGTTGGGCTAACCTCAATACAAAAGATTCTAAATGGGAAAAT<br>GCAAGCTGGGAAGCGGTCGGTAAACCAGGTTCAGGTTCGGCTGATCTGATATACTTCTTAGCAAAT<br>GGTAAAATTGTTGTTCTGCTGAAGGAAGAAGACGGTAAGTATATCATTAAATTATCAACATTTGAAGA<br>CTTCCTGAAAGGAGAAACAATTGTGGAGCTCGAAACGAGTAAAGAGGAAGCCGAAAAACTCATTGA<br>GGAAGCCAAAACCCTGGACGCAGACGGCATTATCGAATTATTTAAGAAAGCTAAAAAGGAATAA
**LptDm15**	ATGTCTGGTTCTCATCATCATCATCATCATAGCAGCGGCATCGAAGGCCGCGGCCGCCAGTACAAA<br>CTGATCCTGAACGGTAAAACCCTGAAAGGTGAAACCACCACCGAAGCTGTTGACGCTGCTACCGCG<br>GAAAAAGTTTTCAAACAGTACGCTAACGACAACGGTGTTGACGGTGAATGGACCTACGACGACGCT<br>ACCAAAACCTTCACCGTTACCGAAAGCAGCGGCGAAAACCTGTACTTCCAGGGGGATACGAACGAT<br>TTGCCAGTAACTATAAATGCTGACCATGCCAAAGGAGATTACCCAGATGACGCAGTATTCACTGGTT<br>CAGTGGATATAATGCAGGGAAACTCTAGATTACAGGCGGACGAGGTTCAGCTGCATCAGAAGGAGG<br>CACCTGGCCAGCCGGAACCCGTGCGTACGGTGGATGCACTTGGGAATGTTCACTATGATGATAATC

	AAGTCATTCTGAAAGGCCCAAAAGGTTGGGCCAATCTGAATACAAAAGATGCACGTTTCGAGAACGC<br>CAGTTATGAAGCAGTCGGAAAACCAGGGAGCGGAAGTGCCGATAAAATTTATTTCCTGGCGAACGG<br>TAAAATAGTTGTGTTAGTTAAGAAGGAGGATGGTAAATGGTATGTGGAGTTGAGTACATTCGAAGAC<br>TTACTGGAAGGCAAAAAATTGGTTTCTCTCGAACTTTCAAAAGAGGAAGCTGAAAAGTTGCTCGAAG<br>AGGCCAAAAAGTTAGATGCGGAGGAAATTATCGAGCTCTTTAAGAAGAAAAAGGAAGAATAA
**LptDm16**	ATGTCTGGTTCTCATCATCATCATCATCATAGCAGCGGCATCGAAGGCCGCGGCCGCCAGTACAAA<br>CTGATCCTGAACGGTAAAACCCTGAAAGGTGAAACCACCACCGAAGCTGTTGACGCTGCTACCGCG<br>GAAAAAGTTTTCAAACAGTACGCTAACGACAACGGTGTTGACGGTGAATGGACCTACGACGACGCT<br>ACCAAAACCTTCACCGTTACCGAAAGCAGCGGCGAAAACCTGTACTTCCAGGGTGATACCAATGAT<br>CTTCCGGTGACTATCAATGCCGACCATGCGAAGGGCGATTATCCTGATGACGCAGTGTTCACCGGT<br>TCGGTGGACATAATGCAGGGTAACTCACGCCTGCAGGCAGATGAGGTTCAACTGCATCAGAAAGAA<br>GCCCCAGGCCAACCTGAACCTGTCCGCACAGTCGATGCCCTCGGTAATGTTCATTATGACGACAAC<br>CAAGTGATATTAAAGGGGCCGAAAGGTTGGGCAAATCTGAACACGAAAGATGCCGTTTTCTATGATG<br>CCACCATTGAAACGGTTGGCGCGCCGGGCAGTGCATCAGCCAAAAAAATCGAGTTCAAAGATAATG<br>GTAAAACGCTCGTTTTGGATAACGCGACATTTAAGTCTTATGAAGAAGCTAAGAGCCTGGTAGAATA<br>TTTTATCAAGAAGATGGGTGTGAAAACCATAATCGTGAATAACGCCACGGGCGCGATTACAGAAGAA<br>CTGAAAAAAGAACTTGAAAAACTGGCAAAAGAACTTGGGGCAAAACTGACTATTACACTGAAGTAA
**LptDm17**	ATGTCTGGTTCTCATCATCATCATCATCATAGCAGCGGCATCGAAGGCCGCGGCCGCCAGTACAAA<br>CTGATCCTGAACGGTAAAACCCTGAAAGGTGAAACCACCACCGAAGCTGTTGACGCTGCTACCGCG<br>GAAAAAGTTTTCAAACAGTACGCTAACGACAACGGTGTTGACGGTGAATGGACCTACGACGACGCT<br>ACCAAAACCTTCACCGTTACCGAAAGCAGCGGCGAAAACCTGTACTTCCAGGGAGACACTAATGAC<br>CTTCCCGTTACGATAAATGCCGACCACGCAAAGGGCGACTATCCCGACGATGCCGTCTTTACTGGT<br>AGCGTTGATATTATGCAGGGCAATAGTCGGCTTCAGGCAGATGAAGTTCAATTACACCAAAAAGAGG<br>CCCCGGGCCAGCCCGAGCCGGTCCGTACGGTCGATGCTTTAGGTAATGTTCACTATGATGACAACC<br>AGGTTATTTTAAAAGGGCCTAAGGGATGGGCGAATCTGAATACCAAAGATTGGGTATTCTACGATGC<br>GTCTATAGAAACTGTTGGAGCGCCTGGCTCTGCTTCGGCCAAAGTAATTAAGAGCTTAGATAACGGT<br>AAAACCCTGGAATTCGATGATGCAACCTTTGATTCGTATGAAAATGCTGAGGCGTTGATCGAACACG<br>CGATAAAGACAATGGGTGTGAAAACCATTACGATAAATAACGCCACCGGCGCGATCACGGCAGAGA<br>AAGCGACGAAACTCAAAGCGAAGGCGGCAGAGCTGGGCGCAACGTTGACCATTACATTGAAATAA
**LptDm18**	ATGTCTGGTTCTCATCATCATCATCATCATAGCAGCGGCATCGAAGGCCGCGGCCGCCAGTACAAA<br>CTGATCCTGAACGGTAAAACCCTGAAAGGTGAAACCACCACCGAAGCTGTTGACGCTGCTACCGCG<br>GAAAAAGTTTTCAAACAGTACGCTAACGACAACGGTGTTGACGGTGAATGGACCTACGACGACGCT<br>ACCAAAACCTTCACCGTTACCGAAAGCAGCGGCGAAAACCTGTACTTCCAGGGTGACACTAATGATT<br>TACCGGTGACTATCAATGCCGACCACGCCAAAGGCGATTACCCTGATGATGCAGTTTTCACAGGCT<br>CAGTTGATATTATGCAGGGCAACAGCCGCCTGCAGGCGGATGAAGTTCAATTACATCAAAAAGAGG<br>CTCCAGGTCAGCCTGAACCAGTGCGTACGGTTGATGCTCTCGGAAACGTCCATTATGATGATAACC<br>AGGTCATTCTGAAAGGACCGAAAGGCTGGGCAAACCTGAACACCAAAGATTGGACTTTTGAAGATG<br>CGTCTATAGAACTCGTTGGCCGGCCTGGTTCCGGTAGTGCGAAAGTAATATCGTCGCTTGATAATG<br>GTAAAAAAGTAAGCTTTGAAGGTCCGGATGGTACGAAAGTGACGCTGACAACTGAAGATGCCTTCAA<br>GATTTTGGAAGAGAACGATGGCGCTGATGCTGAAACGATAGTTAAAGCTGCTAAAGAATATGTAGAA<br>AAGAAATAA
**LptDm19**	ATGTCTGGTTCTCATCATCATCATCATCATAGCAGCGGCATCGAAGGCCGCGGCCGCCAGTACAAA<br>CTGATCCTGAACGGTAAAACCCTGAAAGGTGAAACCACCACCGAAGCTGTTGACGCTGCTACCGCG<br>GAAAAAGTTTTCAAACAGTACGCTAACGACAACGGTGTTGACGGTGAATGGACCTACGACGACGCT<br>ACCAAAACCTTCACCGTTACCGAAAGCAGCGGCGAAAACCTGTACTTCCAGGGAGATACCAATGAT<br>CTGCCGGTCACGATTAACGCAGATCATGCTAAGGGCGATTATCCGGATGACGCTGTGTTTACAGGA<br>AGTGTCGATATAATGCAGGGTAACAGTCGTCTGCAAGCGGATGAGGTCCAACTTCACCAAAAGGAG<br>GCTCCGGGCCAGCCGGAACCCGTACGTACGGTTGATGCGTTGGGGAATGTGCACTATGATGACAA<br>TCAGGTGATCCTGAAAGGTCCGAAAGGTTGGGCGAACTTAAACACTAAAGATGCTGTTTTTTATGAA<br>GTCTCATTTGAAGCTGTCGGACGTCCGGGTTCTGGTTCTGCCGATGTGTTTAAGTTTTTAGACAATG<br>GAGAGAAAGTGGAAGTGGAAATCGGTGATAAAAAAGCGTTGGCGGACAAAGATAAGATTATAGAGT<br>ACGATAGCTTAGAAGCTTTGAAAGCCTTAGCGGCGGCTGCGAAGGCCCTCGGATGGGAGAAAACG<br>GAGAAACTTATTAACGAAAAGATCAAAGAACTGGAATAA
**LptDm20**	ATGTCTGGTTCTCATCATCATCATCATCATAGCAGCGGCATCGAAGGCCGCGGCCGCCAGTACAAA<br>CTGATCCTGAACGGTAAAACCCTGAAAGGTGAAACCACCACCGAAGCTGTTGACGCTGCTACCGCG<br>GAAAAAGTTTTCAAACAGTACGCTAACGACAACGGTGTTGACGGTGAATGGACCTACGACGACGCT<br>ACCAAAACCTTCACCGTTACCGAAAGCAGCGGCGAAAACCTGTACTTCCAGGGAGATACCAATGATT<br>TACCAGTAACGATTAACGCCGATCATGCCAAAGGTGATTATCCAGACGATGCCGTGTTTACCGGATC<br>AGTTGATATAATGCAGGGCAATTCTCGCCTGCAGGCCGACGAAGTACAACTCCACCAGAAGGAAGC

	GCCTGGTCAACCAGAGCCAGTTCGGACTGTTGATGCTCTGGGTAATGTGCATTACGATGACAACCA<br>GGTTATCCTCAAGGGTCCTAAAGGCTGGGCGAATCTTAATACCAAGGATTCTGTTTGGTATGATGTC<br>TCAGCCGAAGTCGTTGGGGAAGAAGGTTCGTTCAGCGCCGAAAAATTCGAAACTCGGAACAATGGT<br>TCTCTTCTGGTTGCCGAAAACGCAAATACGGAGGACTTTATTAAGTTCGCCAAATCCTTAGCTAAAGT<br>TACTGGCAAATCCATTTTGAAGTTTGTCAAAGCTACAAAAGATGGCAAAACACTTAATATCGTGGGTA<br>TTTCAACCGAAGAGGATAAAGAAGCAACACTGGCTGCGATAGAAAAACTTGTGAAGGAAAAAGAAAA<br>GGAAGGCTGGAAAGTAGAAGAAATCAAATAA
**LptDm17_AVI**	ATGTCTGGTTCTCATCATCATCATCATCATAGCAGCGGCATCGAAGGCCGCGGCCGCCAGTACAAA<br>CTGATCCTGAACGGTAAAACCCTGAAAGGTGAAACCACCACCGAAGCTGTTGACGCTGCTACCGCG<br>GAAAAAGTTTTCAAACAGTACGCTAACGACAACGGTGTTGACGGTGAATGGACCTACGACGACGCT<br>ACCAAAACCTTCACCGTTACCGAAAGCAGCGGCGAAAACCTGTACTTCCAGGGAGACACTAATGAC<br>CTTCCCGTTACGATAAATGCCGACCACGCAAAGGGCGACTATCCCGACGATGCCGTCTTTACTGGT<br>AGCGTTGATATTATGCAGGGCAATAGTCGGCTTCAGGCAGATGAAGTTCAATTACACCAAAAAGAGG<br>CCCCGGGCCAGCCCGAGCCGGTCCGTACGGTCGATGCTTTAGGTAATGTTCACTATGATGACAACC<br>AGGTTATTTTAAAAGGGCCTAAGGGATGGGCGAATCTGAATACCAAAGATTGGGTATTCTACGATGC<br>GTCTATAGAAACTGTTGGAGCGCCTGGCTCTGCTTCGGCCAAAGTAATTAAGAGCTTAGATAACGGT<br>AAAACCCTGGAATTCGATGATGCAACCTTTGATTCGTATGAAAATGCTGAGGCGTTGATCGAACACG<br>CGATAAAGACAATGGGTGTGAAAACCATTACGATAAATAACGCCACCGGCGCGATCACGGCAGAGA<br>AAGCGACGAAACTCAAAGCGAAGGCGGCAGAGCTGGGCGCAACGTTGACCATTACATTGAAAGGG<br>AGCGGACTGAATGATATTTTTGAAGCCCAGAAGATCGAATGGCATGAGTAA

Table S6: List of Open Reading Frames (ORF) for LptD constructs. Blue: His-Tag; Yellow: GB1; Purple: TEV Protease Tag; Green: Avitag.
